# Community perspectives on health AI: hopes, concerns and implications for health systems and trustworthy AI

**DOI:** 10.1007/s43681-026-00987-7

**Published:** 2026-02-26

**Authors:** Kerry A. Ryan, Morgan L. Sielaff, Dalya Saleem, Joshua Richardson, Sean Tan, Reema Hamasha, Paige Nong, Sharon L.R. Kardia, Veronica Romanov, Adnan Hammad, Jodyn Platt

**Affiliations:** 1 University of Michigan Medical School, Ann Arbor, USA; 2 University of Michigan, Ann Arbor, USA; 3 University of Minnesota School of Public Health, Minneapolis, USA; 4 University of Michigan School of Public Health, Ann Arbor, USA; 5 Global Health Research, Management & Solutions, Pigeon, Michigan, USA

**Keywords:** Artificial intelligence, Healthcare, Public deliberation, Trust, Health policy, Community participation

## Abstract

The increasing use of artificial intelligence (AI) in healthcare has heightened public focus on issues of trust, transparency, and governance. In this study, we conducted five virtual community deliberations with Michigan residents (n = 159) to explore their hopes, concerns, and perspectives on how to promote trustworthiness in health AI. Participants were predominantly female (65%) with a mean age of 46 years, including 35% African American, 33% White, and 21% Middle Eastern/North African residents, with 40% reporting incomes below $50,000. Participants recognized AI’s potential to improve diagnostic accuracy, efficiency, and access to health information, but voiced concerns about privacy, lack of transparency, diminished human interaction, and insufficient oversight. They recommended clear communication about when and how AI tools are used; regulation and oversight that includes patient and community input; and the use of AI to augment, not replace, human judgment and empathy. These findings informed the development of practice and policy recommendations for trustworthy health AI frameworks emphasizing human-centeredness, transparency, oversight, and accountability, underscoring the critical role of public involvement in AI governance. Future research should engage diverse community perspectives when developing health AI tools to support safe, trustworthy implementation in healthcare.

**Supplementary Information:**

The online version contains supplementary material available at 10.1007/s43681-026-00987-7.

## Introduction

The rapid development and adoption of artificial intelligence (AI) in healthcare has the potential to significantly improve diagnostics, personalize treatment management, and increase operational efficiency [1]. However, the use of AI in healthcare also raises novel ethical issues, including the appropriate use of secondary data, the safety and efficacy of AI tools, privacy, security, and algorithmic bias [2]. The increasing use of AI in healthcare has heightened public attention to the trustworthiness of these tools, their impact on quality of care, and the need for appropriate guardrails to address ethical challenges that AI poses within the healthcare system [3–5]. While many AI tools are ostensibly designed to improve patient care, patients are not well represented in decisions about AI development, procurement, or evaluation [6, 7]. There is also limited understanding of what the public expects when it comes to AI in healthcare.

Recent studies suggest moderate optimism among the U.S. public that AI will improve healthcare overall and increase the accuracy and efficiency of medical diagnoses and treatments [8, 9]. However, patients still prefer that clinicians, rather than AI, make decisions about care [10]. The public also desires greater transparency about the use of AI in healthcare, has low trust that healthcare systems will use AI responsibly, and worries about safety, bias, and over-reliance on AI [7, 11].

Trust and trustworthiness are multidimensional constructs with multiple attributes and requirements, such as competency (i.e., the ability to perform a function with skill and accuracy), fidelity (i.e., prioritizing patient interests), and reliability [12, 13]. Beliefs about trust and trustworthiness are shaped by a set of expectations and past experiences. When these expectations are met, trust is reinforced, but a track record of failures can erode trust. Trustworthy healthcare AI requires not only technological accuracy and reliability, but also responsible development, implementation, governance, and patient-centered practices that address public concerns and align with public values [14].

The current policy landscape that guides the ethical use of AI has significant gaps in protecting patient rights and the public interest. For example, the U.S. Food and Drug Administration’s (FDA’s) scope is limited to AI tools that make treatment decisions without human oversight or are embedded in medical devices [15]. The Assistant Secretary for Technology Policy (ASTP, formerly the Office of the National Coordinator for Health Information Technology) issued rules in 2023 requiring developers of predictive decision support tools to increase transparency by disclosing detailed information about data sources, algorithm development, intended use, fairness, and ongoing validation [16]. In the same year, the National Institute of Standards and Technology (NIST) issued the AI Risk Management Framework that healthcare organizations can voluntarily use to identify, assess, prioritize, and manage AI-related risks throughout the AI lifecycle [17]. Aside from the National AI Initiative of 2020, which established the National Artificial Intelligence Initiative Office, there is no national legislation that establishes regulations or an overarching framework for AI policy and practice. The ability of existing regulatory agencies such as the FDA, ASTP and NIST to develop or enforce AI policy is limited to their legislated domains (and capacity), leaving the majority of AI tools without clear *de jure* or de facto oversight [18]. State, local, or institutional governance may therefore have to fill the gaps using non-standard or ad hoc tools and approaches, thereby raising further questions about public accountability.

While AI technologies are becoming increasingly familiar in everyday contexts such as smartphones and home devices, their use in healthcare remains less well understood by the public [19]. Assessing public comfort, hopes, and concerns about AI in patient care is important for defining expectations of trustworthy AI, and can provide guidance for developing appropriate, evidence-based measures, policies, and safeguards.

Most research on public attitudes about AI is based on surveys and focus groups, which are useful for gathering broad insights into public perspectives. However, given the limited familiarity with and complexity of health care AI, most of these studies do not reflect informed opinions [20]. Public deliberation can bridge this gap by providing more in-depth education to generate substantive dialogues about complex topics, explore trade-offs, and capture diverse viewpoints [21–24]. Virtual meeting platforms now enable deliberative engagement across geographic boundaries while maintaining high-quality, in-depth discussion [25]. In this study, we conducted virtual public deliberations with communities across Michigan to educate participants about the use of AI in healthcare and collect their informed perspectives, hopes, expectations, and concerns to provide public input to inform ethical guidelines and trust-building measures for implementing AI technologies in healthcare.

## Methods

**Fig. 1:**
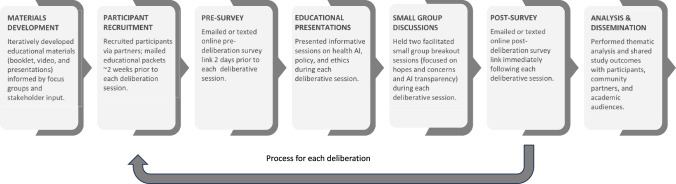
Process the overview of AI in health care deliberations

We conducted five virtual community deliberations in 2024 with members of the lay public living in Michigan (n = 159) to hear their perspectives on the use of AI in healthcare. This study was conducted in partnership with community organizations and approved by the University of Michigan Institutional Review Board and was deemed exempt from federal regulations (HUM00240942). Figure 1 provides a brief overview of our study workflow process from materials development to dissemination.

### Participant recruitment

We recruited participants from various communities across Michigan. For our initial community deliberation, we recruited participants via the UM Health Research website (umhealthresearch.org), which is a platform that helps connect researchers with interested volunteers for University of Michigan research studies. For the other four community deliberations, we collaborated with the Deliberative Engagement of Communities In DEcisions about Resource Spending (DECIDERS) Steering Committee’s (deciders-project.med.umich.edu) community partners and community partner organizations, including Friends of Parkside and the Arab Community Center for Economic and Social Services (ACCESS). Friends of Parkside is a non-profit organization located in Detroit, Michigan, which provides social services for residents of the Villages at Parkside, a public housing community. ACCESS is a Federally Qualified Health Center located in Dearborn, Michigan, rooted in the Middle Eastern and North African (MENA) American community, and providing health, employment, and social services to the broader population within Southeast Michigan. We also worked with three other DECIDERS Steering Committee community partners: a community health educator in the Grand Rapids area (West Michigan) and two community partners serving rural and medically underserved areas in northern and central Michigan. Community partners determined recruitment methods to maximize reach, which included emails, texts, and fliers posted in community centers, food pantries, libraries, newspaper advertisements, and social media. Participation was voluntary, and informed consent (verbal and electronic) was obtained from each participant prior to their participation.

To be eligible for the study, participants had to be 18 years of age or older, fluent in English, living in Michigan, and have access to an electronic device to participate in a virtual deliberation. The study team purposively enrolled participants to ensure diversity in age, education, sex, race, and ethnicity, including intentional recruitment of individuals from underrepresented racial and ethnic groups. To support participation regardless of prior experience with virtual conference software, Zoom training and setup assistance were provided as needed. By conducting the deliberations and surveys online, we facilitated recruitment and participation across a wide geographic area. Participants received $200 for attending the deliberation event and completing both surveys.

### Deliberation overview

Based on a methodology used in previous studies, participants took part in a 5.5-h virtual deliberation session, which included educational presentations, two small group breakout sessions, and an online pre- and post-deliberation survey [23, 24]. See Supplemental Materials for the full deliberative session agenda.

### Deliberation materials

Deliberation materials were developed iteratively by our study team with input from multiple stakeholders. These included two focus groups with the lay public [25], and stakeholder interviews of physicians, academic faculty, healthcare leadership, data scientists, and patients (n = 29). We also held weekly meetings with our community partners (AH, VR) who provided ongoing input related to all deliberation materials, including an educational booklet, expert presentations, surveys, and an educational video.

The educational booklet and expert presentations included an overview of public deliberation, AI and its applications and development in healthcare, types of data used, real-world case studies, and ethical considerations in the use of AI in healthcare.

Approximately two weeks before each deliberation session, participants were mailed a packet that included the educational booklet, instructions for Zoom setup, a handout of presentation slides, a copy of the consent form, and a participant guide with the session agenda. Participants completed 20-min online surveys via Qualtrics (Provo, UT) both before and after the deliberation, accessed through a link sent by text or email. The pre-survey gathered participant demographics, assessed knowledge and comfort with AI in healthcare, trust in healthcare, and priorities for AI transparency and oversight. The post-survey re-assessed trust, comfort, knowledge, and these priorities, and included a session evaluation. Only demographic survey data are reported in this manuscript; other survey findings will be reported separately. The deliberation session agenda, educational booklet, and expert presentations are available in Supplemental Materials. The educational video is available at https://vimeo.com/1071431118?share=copy.

### Expert presentations

Study team members with expertise in public health and learning health systems (JP, SK) gave expert presentations in two plenary sessions. The first plenary presentation provided an overview of the use of AI in healthcare and the associated ethical considerations. The second plenary presentation reviewed the AI health policy landscape and introduced a small group deliberation AI tool label activity. Each presentation was followed by a brief question and answer period. The experts were also available during the breakout sessions to answer any additional questions.

### Small group breakout sessions

For the small group discussions, participants were assigned to one of five breakout rooms, each consisting of six to eight participants and a trained facilitator. Facilitators were selected based on their experience in facilitation or qualitative interviewing. Prior to the deliberation session, facilitators completed a training session that included a detailed review of the session agenda and a run-through of the plenary presentations and the small group discussion activities. Facilitators followed a structured guide for each breakout session. During the first small group breakout session, which is the focus of this report, participants discussed their perspectives on AI and its use in healthcare. Participants were asked about the risks and benefits of AI in healthcare and society, their hopes and concerns, and what additional information they wanted about healthcare AI. In the second breakout session, participants completed an AI tool label prioritization activity; findings from the second breakout session will be reported separately.

### Return of results

To share results back to participants, each participant was mailed a two-page results report following their deliberative session. After all five deliberations were completed, all participants were invited to a 1.5 h virtual town hall where we reported preliminary findings and welcomed input and reactions from attendees. Leaders and experts in AI also attended to learn directly from participants and reflect on how the findings could inform strategies for addressing challenges facing their respective institutions or constituencies. A total of 69 out of 159 deliberative participants attended the town hall (43%), with representation from all five deliberative sessions.

### Analysis

We used descriptive statistics to describe respondents’ demographic and baseline characteristics collected in the online survey. Zoom recordings of the small group breakout session 1 (n = 25 transcripts; 5 deliberations × 5 groups) were transcribed verbatim and de-identified for analysis. To develop our initial codebook, we used both deductive and inductive approaches, drawing on established methods in qualitative research [26–28] with codes based on the small group breakout session 1 questions, as well as insights from facilitator debriefs conducted immediately after each deliberation to capture key observations and emergent issues. For our analysis, we purposively sampled a subset of 10 transcripts (2 per deliberation), aiming for diversity based on small group AI tool label activity results (indicating varying group priorities) and facilitator assignments (to minimize facilitator effect). Two study team members (MS, KR) independently coded these 10 transcripts using MAXQDA (VERBI Software, 2024), iteratively refining the codebook and reconciling discrepancies through consensus meetings. Thematic saturation was achieved within this subset, as no new major themes emerged during coding, supporting the adequacy of this analytic sample. Thematic analysis was led by an experienced qualitative researcher member (KR), with input from the study team, to identify important themes and illustrative quotations. In addition, we used MAXQDA AI Assist’s ‘summarize’ and ‘chat’ features to make direct inquiries (via prompts) within transcripts and previously coded segments to ensure comprehensiveness and mitigate human bias. However, final interpretations, verification, and analyses were made by the study team.

## Results

Across all five deliberative sessions, 173 individuals were enrolled (i.e., agreed to participate and were sent a link to the pre-survey), and 159 (92%) participated by attending the deliberative session and completing both the pre- and post-survey. Our participants were predominantly female (65%) with a mean age of 46 years. The racial composition included 35% African American, 33% White, and 21% Middle Eastern/North African (MENA), and reflected the demographics of the geographic regions and communities served by community partners. Forty percent of participants reported incomes below $50,000, and nearly half (47%) resided in areas classified as high social vulnerability, as measured by the Social Vulnerability Index (SVI) [29]. See Table 1 for full demographics of all five deliberations and in total.

**Table 1:** Participant characteristics (n = 159)

	Deliberation 1	Deliberation 2	Deliberation 3	Deliberation 4	Deliberation 5	Total
	n = 37	n = 35	n = 33	n = 26	n = 28	(% or SD)
*Gender*						
Female	20 (54%)	25 (71%)	23 (70%)	17 (65%)	19 (68%)	104 (65%)
Male	17 (46%)	10 (29%)	10 (30%)	6 (23%)	9 (32%)	52 (33%)
Neither of these describe me	0 (0%)	0 (0%)	0 (0%)	3 (12%)	0 (0%)	3 (2%)
Age, Mean (SD)	44 (16.4)	51 (15.9)	39 (14.7)	46 (16.2)	48 (15.9)	46 (16.2)
*Race/Ethnicity*						
African American or Black	10 (27%)	33 (94%)	0 (0%)	8 (31%)	4 (14%)	55 (35%)
American Indian or Alaska Native Asian American or Asian Hispanic or Latino Middle Eastern or Arab American Pacific Islander or Hawaiian Native White Multi-racial/multi-ethnic	0 (0%) 2 (5%) 1 (3%) 2 (5%) 0 (0%) 16 (43%) 6 (16%)	0 (0%) 0 (0%) 0 (0%) 0 (0%) 0 (0%) 0 (0%) 2 (6%)	0 (0%) 0 (0%) 0 (0%) 31 (94%) 0 (0%) 0 (0%) 2 (6%)	0 (0%) 0 (0%) 1 (4%) 1 (4%) 0 (0%) 15 (58%) 1 (4%)	0 (0%) 0 (0%) 1 (4%) 0 (0%) 0 (0%) 22 (79%) 1 (4%)	0 (0%) 2 (1%) 3 (2%) 34 (21%) 0 (0%) 53 (33%) 12 (8%)
*Highest level of school completed*						
Less than BA	11 (30%)	26 (74%)	10 (30%)	14 (54%)	7 (25%)	68 (43%)
BA	18 (49%)	8 (23%)	16 (48%)	6 (23%)	11 (39%)	59 (37%)
More than BA	8 (22%)	1 (3%)	7 (21%)	6 (23%)	10 (36%)	32 (20%)
*Work in healthcare field*						
Yes	15 (41%)	12 (34%)	18 (55%)	15 (58%)	15 (54%)	75 (47%)
*Household income*						
Less than $50,000 $50,000 to $75,000 $75,000 to $100,000 $100,000 to $150,000 More than $150,000 Prefer not to answer	9 (24%) 7 (19%) 9 (24%) 6 (16%) 4 (11%) 2 (5%)	24 (69%) 0 (0%) 0 (0%) 0 (0%) 2 (6%) 9 (26%)	11 (33%) 10 (30%) 5 (15%) 1 (3%) 2 (6%) 4 (12%)	10 (39%) 6 (23%) 4 (15%) 4 (15%) 0 (0%) 2 (8%)	10 (36%) 3 (11%) 2 (7%) 8 (29%) 4 (14%) 1 (4%)	64 (40%) 26 (16%) 20 (13%) 19 (12%) 12 (8%) 18 (11%)
*Employment status*						
Working Not working (retired) Not working (disabled) Not working (looking for work) Not working (other) Prefer not to answer	26 (70%) 3 (8%) 3 (8%) 0 (0%) 4 (11%) 1 (3%)	13 (37%) 6 (17%) 2 (6%) 4 (11%) 4 (11%) 6 (17%)	27 (82%) 1 (3%) 0 (32%) 2 (6%) 2 (6%) 1 (3%)	17 (65%) 3 (12%) 2 (8%) 0 (0%) 2 (8%) 2 (8%)	14 (50%) 8 (29%) 4 (14%) 0 (0%) 2 (7%) 0 (0%)	97 (61%) 21 (13%) 11 (7%) 6 (4%) 14 (9%) 10 (6%)
*Health status*						
Excellent Very good Good Fair Poor	6 (16%) 10 (27%) 16 (43%) 5 (14%) 0 (0%)	1 (3%) 6 (17%) 10 (29%) 15 (43%) 3 (9%)	2 (6%) 11 (33%) 14 (42%) 6 (18%) 0 (0%)	1 (4%) 8 (31%) 10 (39%) 4 (15%) 3 (12%)	1 (4%) 5 (18%) 15 (54%) 3 (11%) 4 (14%)	11 (7%) 40 (25%) 65 (41%) 33 (21%) 10 (6%)
*Social Vulnerability Index (SVI)*^*1*^						
High: (0.7501–1.0) Mid-high (0.5001–0.7500) Mid-low (0.2501–0.5000) Low (0.0–0.2500)	13 (35%) 3 (8%) 15 (41%) 6 (16%)	29 (83%) 1 (3%) 2 (6%) 3 (9%)	29 (88%) 0 (0%) 2 (6%) 2 (6%)	0 (0%) 9 (35%) 12 (46%) 5 (19%)	0 (0%) 12 (43%) 9 (32%) 7 (25%)	71 (45%) 25 (16%) 40 (25%) 23 (14%)

Rounding effects may result in percentage totals other than 100%

^1^The Social Vulnerability Index (SVI) is based on 16 U.S. Census tract variables from the 5-year American Community Survey (ACS). These variables are grouped into four themes that cover major areas of social vulnerability including: Socioeconomic status, household characteristics, racial and ethnic minority status, and housing type and transportation. The SVI is a tool that combines those four themes and measures the relative social vulnerability of U.S. communities. It uses percentile ranks to score the social vulnerability of a community from 0 (lowest vulnerability) to 1 (highest vulnerability). https://www.atsdr.cdc.gov/place-health/php/svi/index.html#cdc_generic_section_3-methodology

### Perspectives on the trustworthiness of AI in healthcare

During the small group discussions, participants across all deliberations shared both positive and negative views (i.e., hopes and concerns) about the use of AI in healthcare, and offered suggestions on how best to address these concerns to ensure the trustworthy application of this technology. Table 2 summarizes key themes and takeaways from participants’ perspectives, highlighting both their hopes and concerns and outlining actionable implications for healthcare policy and practice. Additional illustrative quotes from the small group discussions are provided in Appendix Table 1 in Supplementary Materials.

**Table 2:** Key themes, takeaways and implications for practice and policy

Thematic Area	Take-aways: Patient Hopes and Concerns	Implications for Policy and Practice	What healthcare organizations can do
Information Access and Efficiency	• Improve timely access to health information for both patients and providers • AI could make clinical encounters smoother and help people get the information they need quickly and easily	• Measure and report improvements in access to health information • Evaluate AI for its impact on how patients and providers access or use information • Ensure that AI improves access to health information for all communities, especially underserved groups, and addresses barriers to information	• Conduct baseline assessments such as surveying patients and providers about current barriers to accessing health information • Collaborate with local partners such as libraries, schools, or faith-based organizations to extend information on AI-driven health communication tools and resources • Host community forums and invite feedback about what barriers remain and what AI tools seem most or least helpful
Advancing Clinical Care	• AI has the potential to increase accuracy and speed of diagnoses, allowing for quicker, more accurate treatment • AI could help detect conditions that are often missed and enable medical advances for future generations • AI can assist with ongoing monitoring of patients’ health and clinical status to support timely interventions	• Demonstrate improvements in health outcomes • Ensure AI tools reliably support accurate diagnosis, treatment decisions, and real-world clinical needs, using ongoing evaluation and monitoring to minimize errors • Support AI tools that enable earlier detection of disease and assist with ongoing monitoring of patients’ health	• Form joint task forces for pilot studies on new AI tools and their effect on diagnosis and treatment • Share success stories by publishing case studies showing evidence-based improved outcomes
Streamlining Administrative Processes	• AI can help reduce long wait times, improve appointment scheduling, and lessen paperwork for patients and staff • More efficient healthcare visits and administrative processes due to AI	• Measure and report on patient-centered improvements in healthcare operations (e.g., reduced wait times, satisfaction with administrative tasks) • Evaluate AI for its impact on efficient care delivery, including reduced wait times and smoother clinical encounters	• Use simple kiosks, paper surveys, and mobile tools to get patient input on wait times and satisfaction after encounters involving AI tools • Share improvement dashboards
Transparency	• Participants want to know when and how AI is used in their care as well as how decisions are made • Access to information about how AI works—its purpose, risks, benefits, and the people responsible for it	• Provide clear, up-to-date information about how AI is used in patient care and healthcare operations • Clearly inform patients about how and why AI is being used in their care, what decisions are made by AI, and what the potential benefits and risks are • Provide accessible education so everyone can understand these systems	• Produce multilingual patient brochures or short videos explaining AI’s role in their care • Designate staff as a contact person for questions or concerns about AI. In certain health systems, an embedded ethics team can serve this role • Publish annual community reports explaining current AI uses and upcoming changes, including AI governance practices
Oversight & Regulation	• Participants emphasized the importance of clear policies and accountability for AI use in healthcare • They called for strong oversight, rigorous testing, and ways for community members to be involved in decisions about AI governance	• Create AI governance and communicate practices to patients • Regularly review AI tools with meaningful input from diverse patients and community members to ensure accountability • Develop clear governance, feedback, and redress pathways • Mandate regular audits of AI tools to assess fairness, equity, and bias, with transparent reporting and corrective actions	• Establish a committee or group to regularly review and adopt AI governance practices and include patient representatives as part of the committee • Provide anonymous forms online and in clinics to report problems or concerns regarding AI
Human Role	• AI should complement, not replace, human clinicians; participants value the unique empathy and judgment that only humans provide • Final decisions in care should always rest with patients and healthcare providers, with AI as a supporting tool	• Develop policies stating that clinical decisions should be made by healthcare providers and patients, with AI serving only a supportive role • Integrate regular assessments of AI’s impact on clinician-patient relationships into quality assurance processes	• Adopt written policies that clarify AI is a tool, not a decision-maker • sOffer regular workshops and updates to help clinicians integrate AI safely and effectively with patients

### Hopes for AI: improving accuracy, responsiveness, and efficiency

Participants expressed optimism about AI’s potential to improve many aspects of healthcare in the future. They hoped that AI could support both clinical and administrative tasks, enhance information access, and improve accuracy and efficiency in healthcare. AI was seen as a technology that would soon become commonplace: "In 10 years, it’s going to be something like a calculator, like the internet, like our cell phones…" (Deliberation 3) Participants were excited about AI’s potential to transform healthcare for future generations: "I’m excited because of the groundwork that’s being put in now around AI, and what it could possibly look like for my grandchildren and my great-grandchildren." (Deliberation 1) They also looked forward to AI contributing to important healthcare advances: "Cures that are developed through the use of AI… that would be wonderful." (Deliberation 2).

Participants hoped that AI would allow for efficient and convenient access to health information: “For me, the excitement is just the access to the amount of information that potentially could be helpful […] It’s just available so quickly…" (Deliberation 1) One participant pointed out that AI could enhance clinic visits by providing real-time information: "A lot of doctors carry phones with them during the visits. I had a lot of questions that [the doctor] didn't have the answers for at the time, and he was able to go onto his device and give me a lot of answers." (Deliberation 4) Efficient access to information could also leave more time for doctor-patient communication: "It makes for a more efficient visit from the doctor because they get all the information they need up front […] it does create more space for there to be more time for individual concerns." (Deliberation 5).

Participants saw the potential for AI to improve clinical care, particularly in areas such as diagnosis, prediction, and monitoring. They were hopeful that AI could help providers make quicker diagnoses: "Not only can it detect diseases perhaps a doctor typically couldn’t detect, but could do it much quicker, too” (Deliberation 3), including for underdiagnosed diseases: “I believe there are several diseases that are underdiagnosed by doctors. Something like this might be able to better diagnose based on the statistical information.” (Deliberation 1) They also expressed hopes that AI could increase diagnostic accuracy: “I believe if they collect enough data, it probably can decrease the human error to diagnose the disease.” (Deliberation 1).

Participants hoped that AI would improve monitoring and responsiveness: "[AI could] pick up on something and alert the nursing desk immediately quicker than a nurse actually monitoring themselves," (Deliberation 5), as well as by "flagging" certain conditions, like a family history of cancer, to enable earlier screenings. (Deliberation 3) Participants also hoped that AI could help overcome human limitations: "Things about the healthcare team that are deficient can be overridden by the AI…no matter how tired the doctor is, no matter how old, or a language barrier, other things like that might impact their decision." (Deliberation 2).

Participants hoped AI could streamline administrative tasks, such as scheduling appointments, generating healthcare documents, and reducing patient wait times. AI was seen as potentially beneficial for claim processing, scheduling reminders, and streamlining referrals. Some participants shared experiences of long wait times in healthcare settings: “…sometimes we sit in the waiting room for 2 or 3 h and [doctors will] be in the room with their patients a long time” (Deliberation 4) and expressed hopes that AI could help address this: “I remember the last time I was in the emergency room, it took me five hours to get in. I’m excited to see how fast they can get the appointments going or how long the waiting time is going to be with the use of AI.” (Deliberation 3).

### Addressing concerns and building trust in AI systems

While participants recognized AI’s potential to improve healthcare, they also voiced important concerns. They worried about AI being used without patient knowledge or consent, and about insufficient oversight and accountability for potential errors or harm. They highlighted the importance of preserving human interaction, fearing that increased reliance on AI could diminish the personal connection in care. To build trust, participants called for transparency about how AI is developed and used, clear regulation and oversight, and meaningful involvement of diverse patients and community members in decisions about healthcare AI. Finally, participants wanted to ensure that the human touch was maintained with AI being used as a tool to assist, but not replace, healthcare providers.

#### Transparency

Across all deliberations, participants wanted transparency about the use of AI in their healthcare: "The first thing we would need is transparency. Like some sort of information right upfront on what's being used, why it's being used." (Deliberation 4) They stressed the importance of clear and accessible information about its benefits and limitations: "One of the ways that we can address people’s concerns is just by making sure that there is a lot of clear information about how AI is being used and how it’s being effective, but then also being transparent about potential risks." (Deliberation 1) Participants wanted more detailed information on how AI tools are developed and used, and who is involved in the development process. They sought "transparency of algorithms" and "transparency of the development of the software,” as well as transparency in the data collection process. (Deliberation 4) As one participant explained: "My concerns could be addressed by full transparency. How is it developed? What is the purpose? What is the goal? Who is regulating?” (Deliberation 1).

Some participants also requested clear communication and better AI education to increase public awareness about the use of AI in healthcare. They were concerned that many people were unaware of AI’s widespread use, and they wanted more accessible information about AI “so that the general public can get more educated." (Deliberation 5) Questions were raised about whether patients would be informed about "AI accessing important medical records" and would receive education on "what all of this means and how it's going to be a benefit and a risk to them." (Deliberation 4).

#### Regulation and Oversight

Participants were concerned about the apparent lack of human oversight and regulation around the use of AI in healthcare. One participant likened it to the Wild West: "That’s alarming that there are no policies or laws restricting or really wrapping its arms around AI yet. Anything goes, like it's the wild, wild West." (Deliberation 2) Others stressed the importance of knowing “who regulates the person who creates the algorithm” (Deliberation 5) and of knowing “who’s controlling the people that’s controlling AI?” (Deliberation 2).

Participants called for rigorous testing and oversight to ensure the accuracy of AI in healthcare. They desired more research to build trust in the system and to "really make sure that there [are] no mistakes before you just straight up integrate [healthcare AI]." (Deliberation 3) Participants shared experiences with new technologies like GPS not working well and pointed out that the stakes are higher with healthcare AI: "…people's lives are on the line. If I get lost in the woods, I might be okay. But if a health decision is made poorly, or if it's not caught in time, people can get hurt.” (Deliberation 4) They also wanted accountability and to know what their rights are if something goes wrong, asking for "some kind of precedent or very explicit law or regulation about […] who is responsible and clearly spelling out what kinds of things they can do with it and what rights the people have to opt in or out and if something goes wrong." (Deliberation 1).

Additionally, participants emphasized the importance of involving patients, communities, and individuals like themselves in AI development and decision-making processes. They noted that including public voices was necessary to ensure that regulation and oversight reflect diverse perspectives and protect patients. One person stated, "I just think we should be heard and considered…" (Deliberation 2) Another participant highlighted the need for the public to be "a part of the process of implementing laws and policies concerning AI and our protection." (Deliberation 2) Community input was seen as necessary for building trust: "If we’re not a part of that process, then I don’t think we’re going to trust it completely […] just knowing that there are people like us on the team […] that’s really helpful." (Deliberation 3).

#### The human touch in healthcare

Participants wanted AI to be used as an assistive tool to augment, rather than replace, healthcare providers. They felt that healthcare decisions should be made by human providers and patients, not by an AI tool: "My biggest concern again is the decision-making it has. […] I think the way it should be addressed is that the patient, or whoever is representing them, is always making the final decision." (Deliberation 1) Participants were also concerned that over-reliance on AI could undermine human connection and trust: “You shouldn’t just develop a great AI model and just completely depend on it because then that lack of human trust, that lack of human relationship […] we don’t want that.” (Deliberation 3) They emphasized the need for continued human review, as AI is not yet fully reliable: “A human has to be there just catching little mistakes, especially [with] something still in the process of developing. It’s not a hundred percent there yet.” (Deliberation 3).

AI was seen as helpful for providing a second opinion or as an additional tool in a provider's toolkit: “It can act as a second opinion instead of just being the main opinion. That’s something that I could be excited about.” (Deliberation 1) AI was described as "a resource in the doctor's arsenal of equipment to administer appropriate and good care to his patients." (Deliberation 2). As an assistive tool, AI was perceived as improving human accuracy: “AI can benefit as a complementary tool, not a substitution because being human means, at some points in time, some mistakes are inevitable.” (Deliberation 1).

Context also mattered, particularly when distinguishing between clinical and non-clinical applications. One participant discussed how autonomous AI could enhance efficiency in administrative tasks like claim processing, but noted that it raises liability concerns when making significant clinical decisions: “The question that everyone's having is, should we listen to the AI tool, or should I listen to my training?" (Deliberation 4) Another participant thought it would be “great” if AI reduced patient wait times, but relying on it for diagnosis requires more scrutiny: “[if] I am going to a physician who is incorporating AI to help him with diagnosis, I’d want a second and third opinion.” (Deliberation 3).

Finally, participants wanted to preserve the ‘human touch’ in healthcare, valuing human interaction and empathy: "I think the human element is always important, though, because you just can't go through life looking at facts and coming to a conclusion. I think you have to have some reference to your experience in life yourself." (Deliberation 2) Human empathy and creativity were seen as particularly important in sensitive healthcare situations: “When a doctor’s delivering bad news to a patient, the human empathy should be there. The human creativity should be there." (Deliberation 3) Participants stressed that AI should remain assistive to preserve the human touch: "I’m not against AI at all, but it should stay assistive. If you delete the human interaction, you’re going to delete the whole humane aspect. Medicine is a practice that has to do with human interaction." (Deliberation 3).

## Discussion

In this study, we conducted five community deliberations throughout Michigan and found a range of hopes and concerns for AI in healthcare. Participants expressed optimism about AI’s promise to address inefficiencies, improve access to information, and advance clinical care in the U.S. healthcare system. They hoped that AI could support more accurate diagnoses, streamline administrative tasks, and improve access to health information for both patients and providers. At the same time, participants stressed the importance of preserving human connections in healthcare and that AI should be used to assist, not replace, clinicians in decision making. Participants called for greater transparency, including being informed about when and how AI is used in healthcare, and emphasized the importance of oversight and accountability mechanisms to support the development of trustworthy healthcare AI.

The hopes and concerns voiced by community members during these deliberations highlight the need for healthcare AI policy priorities that are patient-centered, transparent, and aligned with public values as AI becomes more integrated into healthcare delivery. These discussions offer valuable guidance by identifying priorities and attributes that future guidelines should incorporate, drawing directly from those whom AI tools are meant to serve and who may be at greatest risk of harm from unregulated or poorly designed technologies. This focus also aligns with broader concerns that many AI tools are trained on datasets that do not fully reflect the racial, ethnic, linguistic, and cultural diversity of U.S. populations, and often lack culturally sensitive design, limiting their effectiveness and acceptability for patients from diverse backgrounds [30, 31]. As a result, issues of bias, lack of accuracy for underrepresented groups, and the “black box” nature of AI decision-making remain especially salient, particularly for communities with histories of exclusion or discrimination in healthcare [32]. Our findings are also consistent with the Ada Lovelace community deliberation initiative in the UK, which found that "AI for public good" is a values-based concept where AI can only support the public good when it harmonizes with core public values [33].

These factors underscore the importance of advancing transparency, cultural responsiveness, and robust community engagement to promote trustworthy AI in healthcare. Table 2 presents key policies and practices for trustworthy AI in healthcare that directly reflect the priorities, expectations, and values shared by our participants. To our knowledge, this is the only published set of policies and practices developed through direct community engagement with patients and the public. These can help guide policymakers and healthcare leaders in developing AI that is responsive to the needs of patients and communities.

Translating these policy recommendations into practice will require concrete steps within healthcare systems to foster trust and maximize the benefits of AI. Participants identified the need for public- and patient-facing resources, which could take the form of AI labels, educational materials (such as webinars, booklets, social media, and organizational websites), and informed consent processes that disclose the risks and benefits of AI tools in plain language. These resources and materials should also be culturally tailored, linguistically accessible, and designed for broad patient education to ensure information resonates across diverse communities. We continue to collaborate with our community partner organizations to develop education and training programs on healthcare AI for healthcare staff and community health workers. These training initiatives are being co-designed with community partners based on the priorities and concerns identified during our deliberations. Providing communities with clear, relevant, and understandable information is key to building the trust needed to support ethical AI adoption (safe, private, secure, etc.). Healthcare organizations should develop accessible ways for patients to share feedback and voice concerns about how AI is used in care. At the same time, vendors and policymakers should prioritize engaging with patients and communities to ensure that patient input shapes the ongoing development, deployment, and oversight of these technologies, reinforcing the goals of a patient-centered and learning health system.

Methodologically, public deliberation addresses several gaps in current literature about public perspectives on the use of AI in healthcare. Frost et al. (2024) found that lack of prior AI knowledge among patients was a common limitation in studies of public views on healthcare AI [20]. Our deliberative design addressed this gap by integrating educational materials with facilitated discussions to elicit informed participant perspectives. Calls for community engagement in healthcare AI point out that such engagement should be continuous, diverse, and embedded throughout the AI lifecycle [34]. Our structured deliberative process, ongoing community partnerships, and co-designed training programs model such a sustainable engagement approach. AI best practices call for authentic engagement with patients and communities, as well as standards that promote data quality, transparency, and diversity [30, 35]. Our community deliberations operationalize these principles and practices by centering diverse voices in defining expectations for trustworthy AI and by articulating what adequate representation and acceptable data use should look like from community perspectives.

This study has several limitations to note. Although we engaged community members across the state of Michigan and included perspectives from communities often underrepresented in research, our qualitative, community-based approach means that our findings are not necessarily representative of the general public. Our participants were also predominantly female, which may have influenced the results. Future studies should evaluate the generalizability of the priorities we identified using large, population-based surveys. Expanding this deliberative approach nationally would also help capture broader geographic perspectives and further validate these community-informed policy recommendations. In addition, all deliberations and materials were only available in English, which excluded potential non-English speaking community members**.** Further research is needed to capture perspectives from non-English-speaking populations, given likely differences in access to care, data representation, and experiences of discrimination. Offering deliberations in non-English languages could reveal a broader range of hopes and concerns. The scope of this analysis is also limited to the first breakout session, which explored participants' general perspectives on AI and its use in healthcare. Additional insights from the second breakout session on AI tool label prioritization and survey results will be reported in future work. Finally, the significant time commitment of attending a 5.5 h deliberation may have prevented some individuals from participating.

## Conclusion

This study identified key hopes and concerns voiced by community members regarding the integration of AI into healthcare. Participants highlighted AI’s potential to improve efficiency, accuracy, and access to information, while also raising concerns about preserving human connection, ensuring transparency, and securing meaningful oversight and accountability. These insights can inform the development of practice and policy recommendations for trustworthy and effective healthcare AI policy frameworks that reflect public values. Based on our findings, these frameworks should emphasize human-centeredness, transparency, oversight, and accountability. By engaging communities in these deliberations, our work underscores the importance and impact of meaningful public involvement in shaping AI governance. Future research should continue to engage broad and diverse perspectives when developing and evaluating AI governance and communication tools, including patient-facing labels, notification processes, and public education about AI to support safe and trustworthy implementation of AI in healthcare.

## Supplementary Information

Below is the link to the electronic supplementary material.

Supplementary file1 (PDF 7407 KB)

## Data Availability

The deliberation session agenda, educational booklet, expert presentations, and supplementary quotes are available in Supplemental Materials. The raw qualitative data are not publicly available due to privacy or ethical restrictions.
