# Supplementary material for: Community perspectives on health AI: hopes, concerns and implications for health systems and trustworthy AI
Source: AI Ethics. 2026 Feb 26;6(2):176. doi: 10.1007/s43681-026-00987-7 (PMC12945899; doi:10.1007/s43681-026-00987-7)
Supplement: Supplementary file 1 — Supplementary file1 (PDF 7407 KB) [file 43681_2026_987_MOESM1_ESM.pdf]

Virtual Session Agenda

Time	Duration	Activity & Topic
9:00am – 9:10am	10 min	**Logging On**<br>▪ Participants, please log-in promptly at 9:00am to give time to fix any computer glitches!
9:10 – 9:30am	20 min	**Welcome and Introductions**<br>▪ Introductions<br>▪ Overview of deliberation day
9:30 - 10:15am	45 min	**Plenary Session 1: AI in Healthcare**<br>▪ What we are going to do today & why<br>▪ What is AI?<br>▪ How is it used in healthcare?<br>▪ What are the ethical concerns?
10:15 – 10:25am	10 min	**Q & A session**<br>▪ With Plenary Session 1 presenters
10:25 – 10:30am	5 min	**Break**
10:30 – 11:30am	60 min	**Small Group Icebreaker & Discussion**<br>▪ Brief introductions & Icebreaker<br>▪ Perspectives on AI<br>▪ Hopes & Concerns<br>▪ Things you want to know about AI tools
11:30am – Noon		**Break for Lunch**
Noon – 12:05pm	5 min	**Logging On**<br>▪ Participants, please log-in promptly at Noon to give time to fix any computer glitches!
12:05 – 12:35am	30 min	**Plenary Session 2: Policy and Notification for AI**<br>▪ AI Policy<br>▪ Introduction to AI Label activity
12:35 – 12:45pm	10 min	**Q & A session**<br>▪ With Plenary Session 2 presenters
12:45 – 1:45pm	60 min	**Small Group Session: AI Label**<br>▪ Creating individual AI labels<br>▪ Working together to create small group AI label<br>▪ Modifications and discussion
1:45 – 1:50pm	5 min	**Break**
1:50 – 2:30pm	40 min	**Large Group Review**<br>▪ Facilitators share key take-aways from small groups<br>▪ Large group discussion<br>▪ Wrap-up/feedback

The Use Of Artificial Intelligence And Smart Technology Tools In Health Care: A Public Deliberation

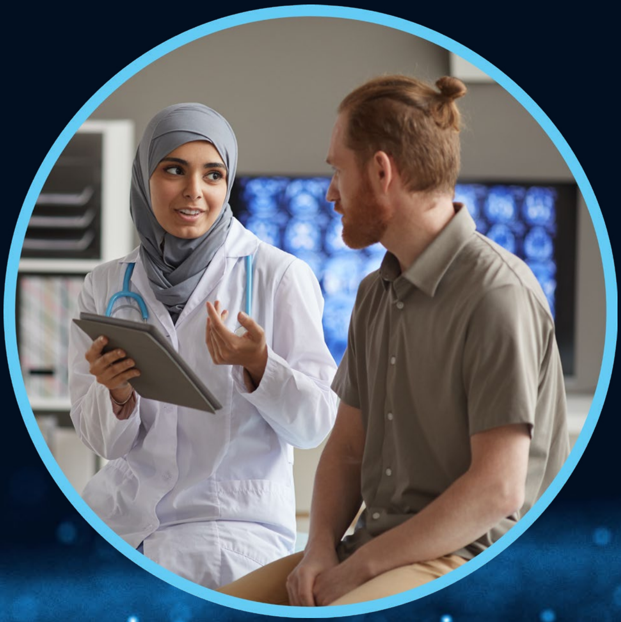

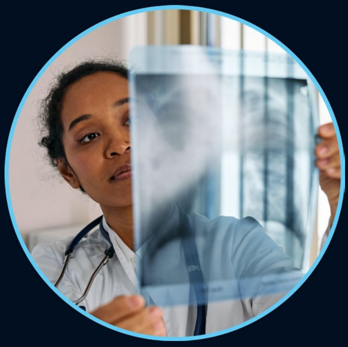

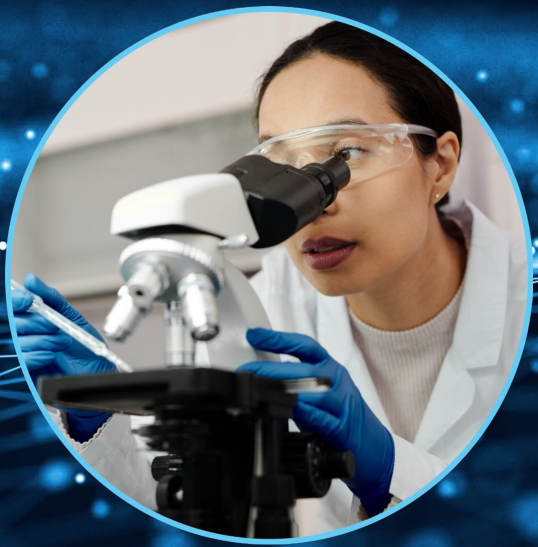

UNIVERSITY OF MICHIGAN MEDICAL SCHOOL
MICHIGAN MEDICINE

TABLE OF CONTENTS

**Welcome!**	2
What is a Public Deliberation?	2
Deliberation Overview	2
**What Is Artificial Intelligence?**	3
**How Is AI Used In Healthcare?**	4
**How Are AI Tools Made?**	6
**What Types Of Data Do AI Tools Use?**	8
**Use Case: Olivia Tan**	9
**Do AI Tools Work?**	10
**Who Uses AI Tools In Healthcare?**	12
**Cause For Hope Or Concern?**	14
**Where Is This Headed?**	18
**What Does The Public Need To Know?**	20
**Glossary Of Terms**	22

WELCOME!

Thank you for participating in our public deliberation about artificial intelligence (AI) and smart technology in healthcare. We hope to hear from you:

- **What are your hopes and concerns about the use of AI in healthcare?**
- **What are the key pieces of information that the public needs to know about the use of AI in healthcare?**

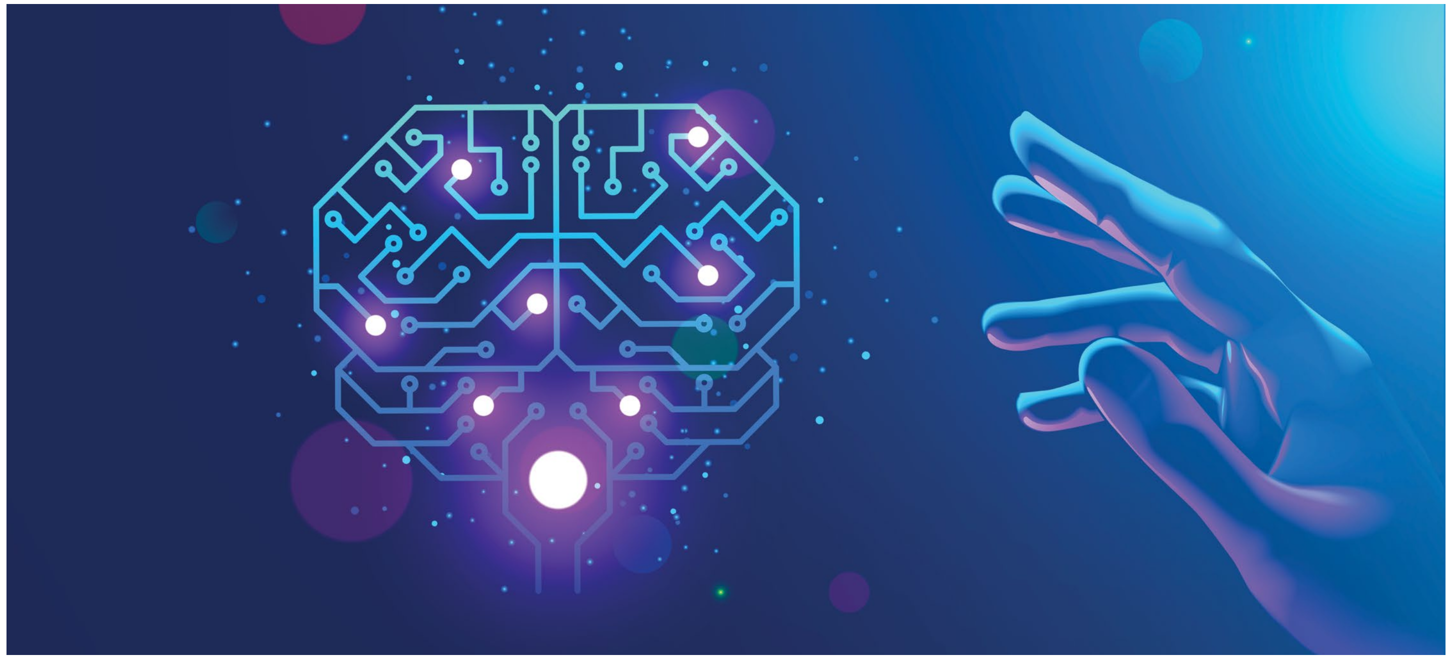

What is a Public Deliberation?

Public deliberation is a community discussion that brings people into the process of making decisions or solving problems that affect them. In the deliberation, we work together to understand the challenging issues and consider different perspectives. For this project, we will discuss the use of AI in healthcare.

Deliberation Overview

During the deliberation, you will hear about the use of AI tools in healthcare from experts in this area. *You are not expected to be an expert*, but we would like you to bring your opinions, values, and ideas to the conversation. You will work with other participants to make recommendations that can shape policies and best practices for the use of AI in healthcare.

WHAT IS ARTIFICIAL INTELLIGENCE?

Artificial intelligence, or AI, uses large amounts of data to process information, to make predictions, automate processes, or help people make decisions. AI is used in many aspects of life - it is used in “smart” technologies or smart devices like Siri, Alexa, or GPS navigation in your car or on your phone. Stores use AI to see what you buy, and then send you coupons or advertisements.

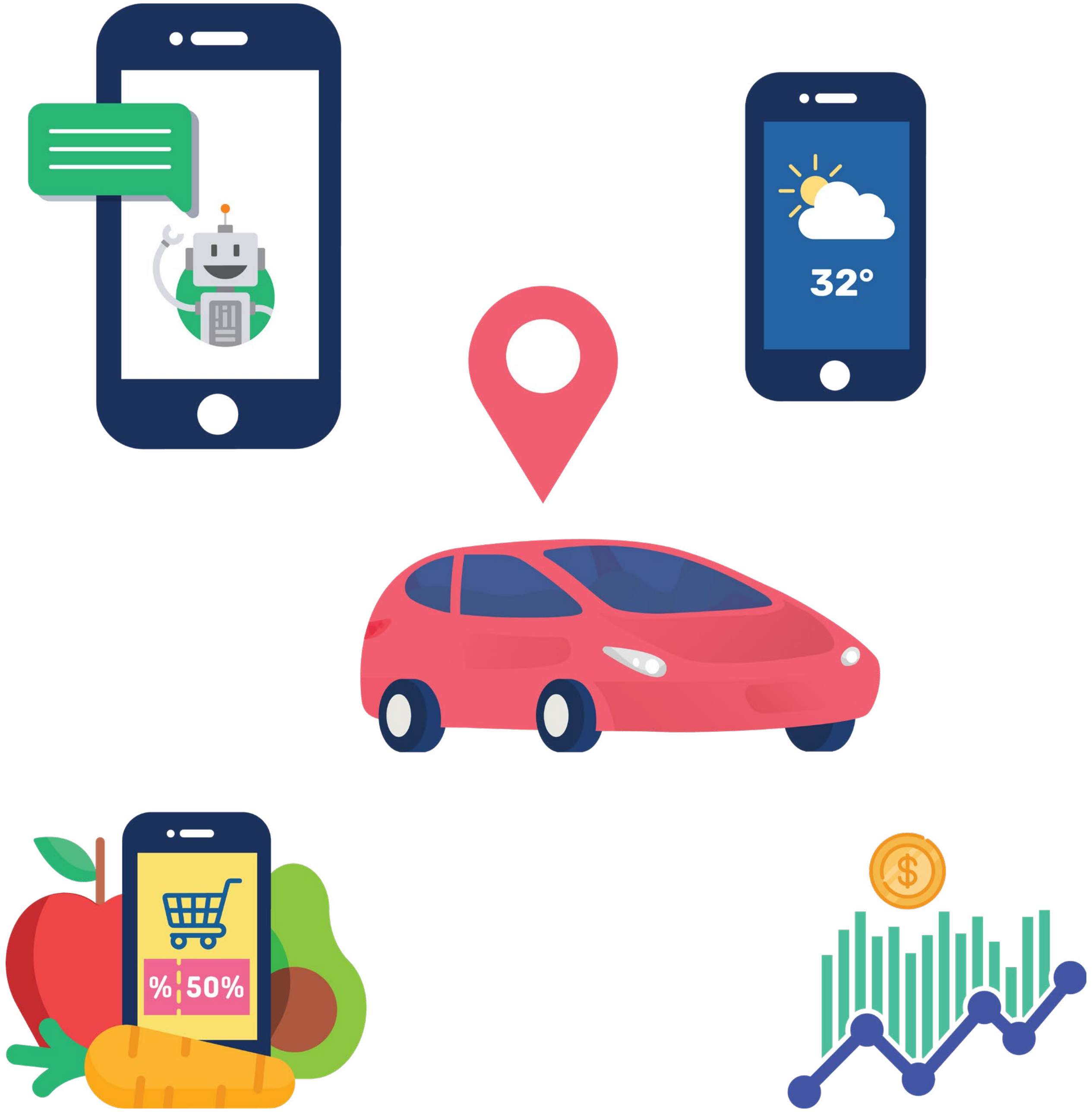

HOW IS AI USED IN HEALTHCARE?

AI tools in healthcare have clinical uses, administrative uses, or personal uses.

In **clinical care**, AI tools may:

- Predict your chance of developing a disease
- Read images to detect cancer or other diseases
- Recommend treatments
- Help diagnose diseases
- Be used in monitoring devices like insulin pumps or heart pacemakers

AI can also have **administrative uses** in healthcare. AI tools may:

- Help scheduling patient appointments
- Transcribe doctor visit notes
- Automate medical billing
- Draft email communications to patient
- Manage inventory of medicines and medical supplies

There are also **personal uses** of AI tools for health. For example, apps on smart phones or smart watches can:

- Track medications
- Send health alerts
- Monitor your heart rate, exercise, or sleep pattern

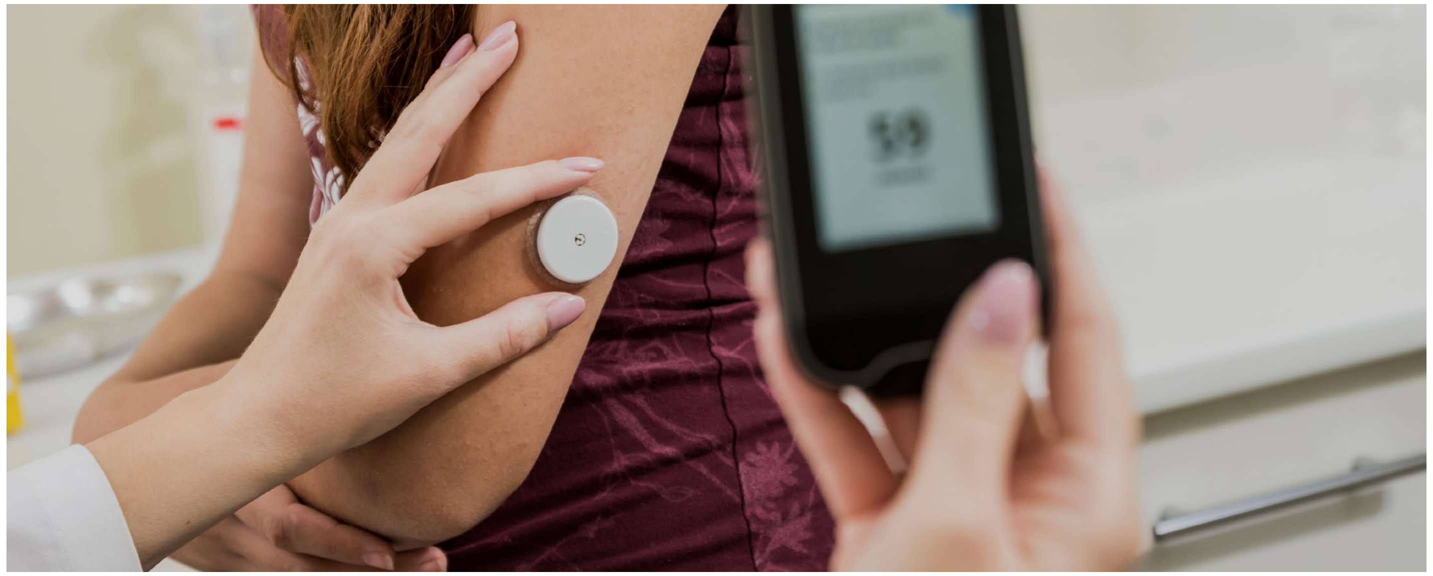

Clinical use: monitoring insulin pump

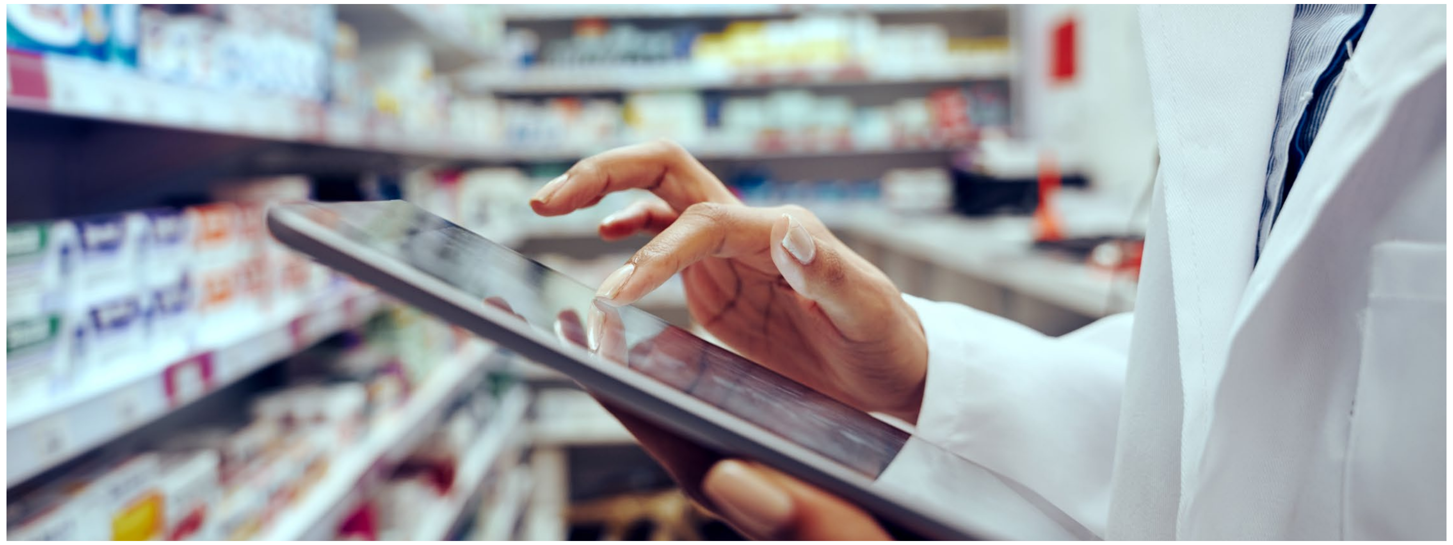

Administrative use: managing medical supply inventory

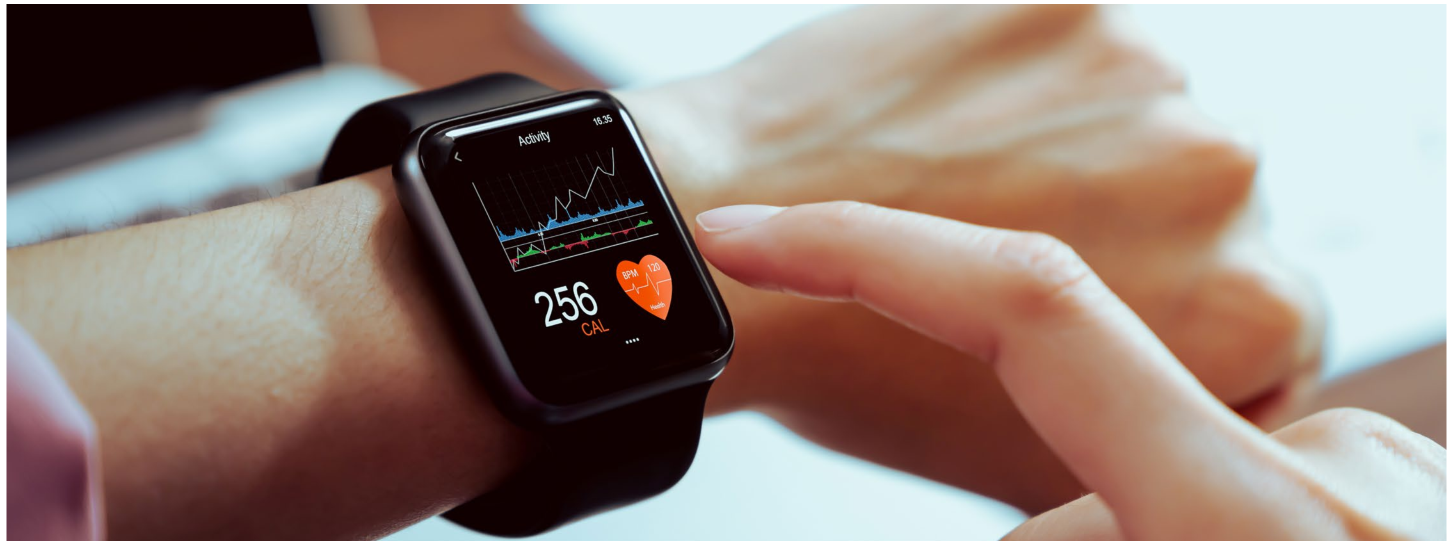

Personal use: monitoring heart rate and exercise

HOW ARE AI TOOLS MADE?

AI tools are created by training computers to recognize patterns in large amounts of data, like images or text. The computer is given many examples, tested on how well it performs, and then given feedback so that it can improve. As the computer learns from more data, it gets better at tasks like understanding human language or recognizing objects in pictures.

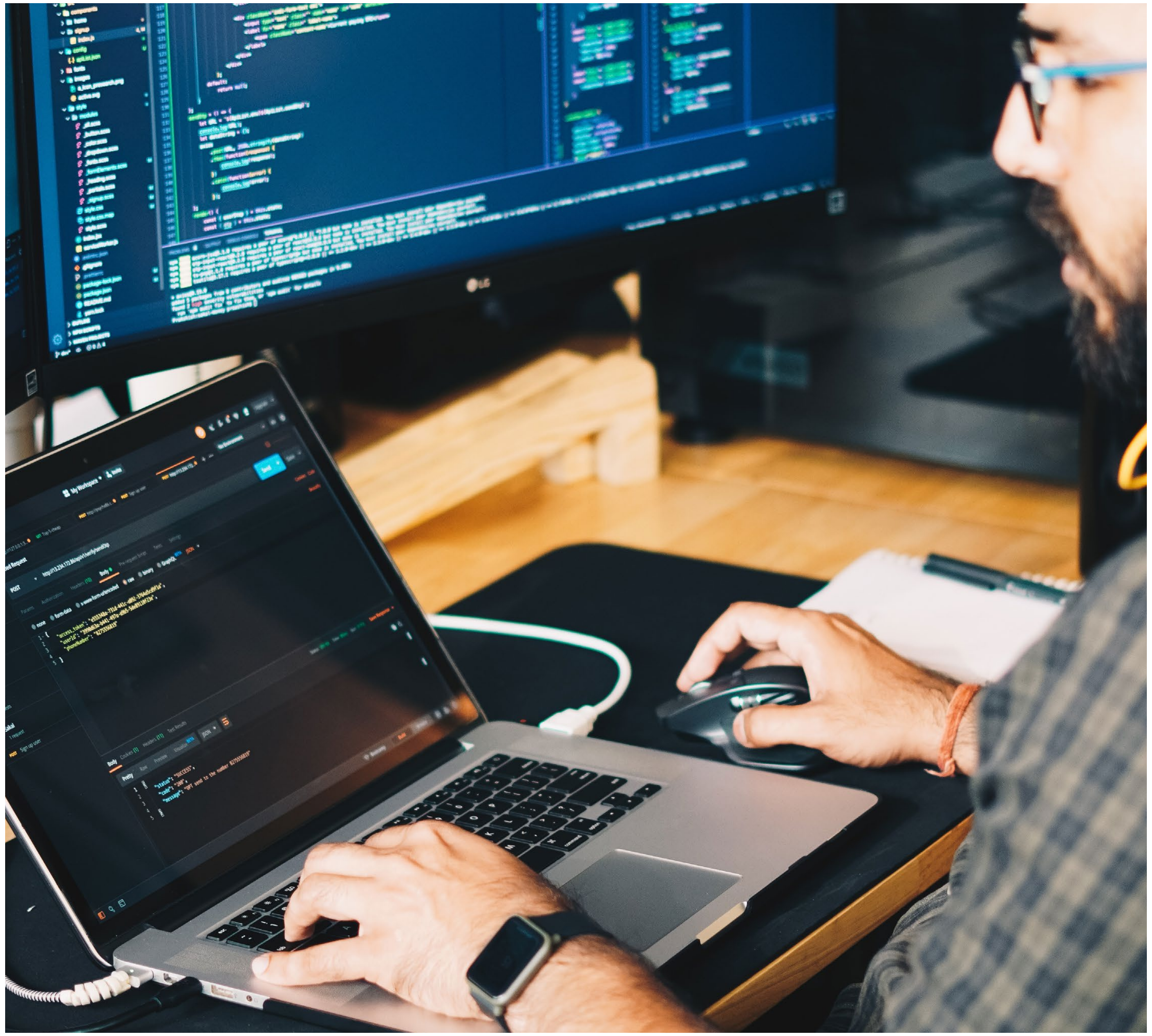

A programmer writing code on a computer

For instance, an AI tool can be used to diagnose diabetic retinopathy, a condition where high blood sugar damages blood vessels in the eye. The AI tool is developed and tested using datasets with thousands of pictures of eyes that have already been labeled as healthy ("negative" for the disease) or unhealthy ("positive" for the disease).

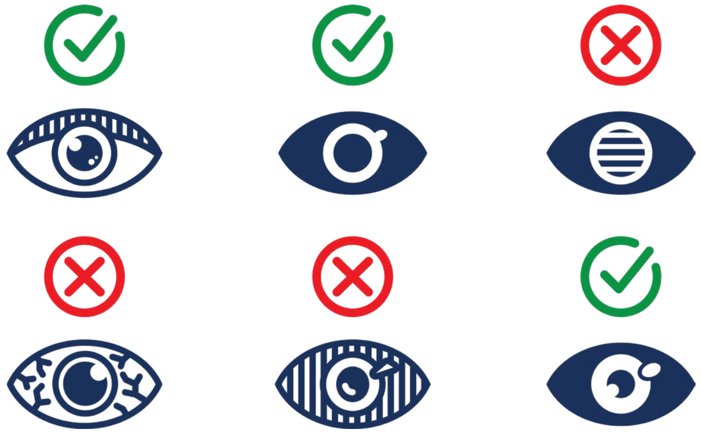

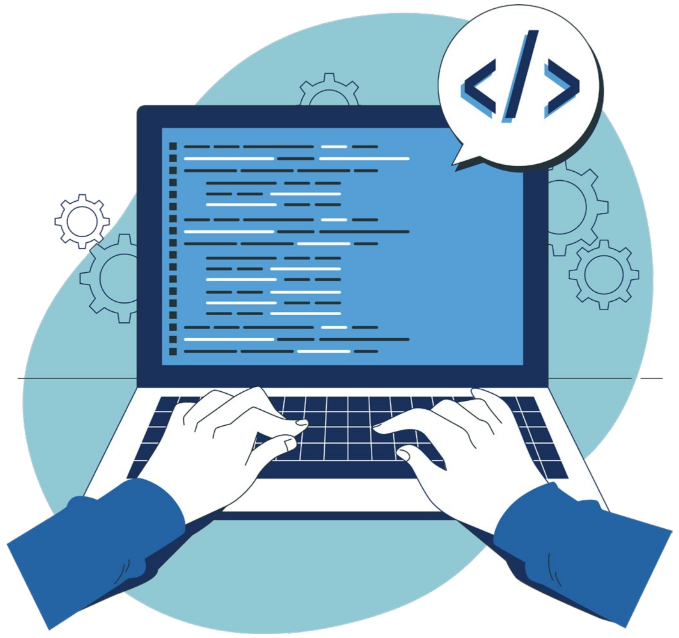

After the tool has been tested, a doctor can input a new picture of an eye from a patient who needs a diagnosis. The AI tool will provide an answer about whether diabetic retinopathy is present based on what it learned from the original dataset of images.

The AI tool will continue to use data from images, patient diagnoses, and outcomes to improve how accurate it is over time.

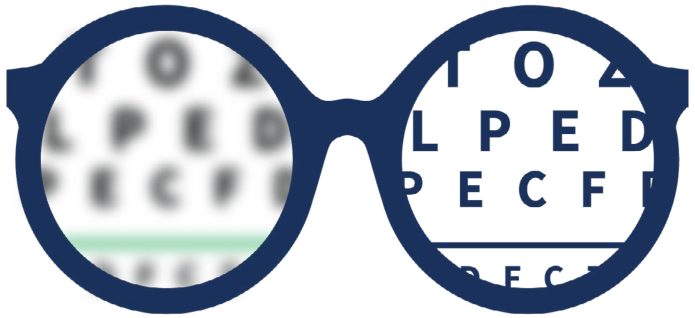

WHAT TYPES OF DATA DO AI TOOLS USE?

In general, having more data helps AI tools to make more accurate predictions. The specific types of data an AI tool may use depends on its particular purpose.

For instance, suppose your health system wants to develop an AI tool that better predicts patient risk of throat cancer in order to improve treatments for people with high risk of cancer. The tool may use information about:

Lifestyle / behavior

- Recreational drug use
- Alcohol
- Smoking status
- Diet and nutrition
- Physical activity

Demographics

- Sex
- Age
- Race
- Ethnicity
- Disability status

Medical History

- History of sexually transmitted disease
- Family history of cancer
- Personal history of cancer
- Genetic information
- Immunization record
- Lab tests
- Prescription drugs
- Weight
- Height
- Personal history of cancer
- Other health problems

Social Determinants of Health

- Zip code
- Transportation insecurity
- Food insecurity
- Income insecurity
- Employment insecurity
- Personal safety
- Environmental exposures
- Work history

USE CASE: OLIVIA TAN

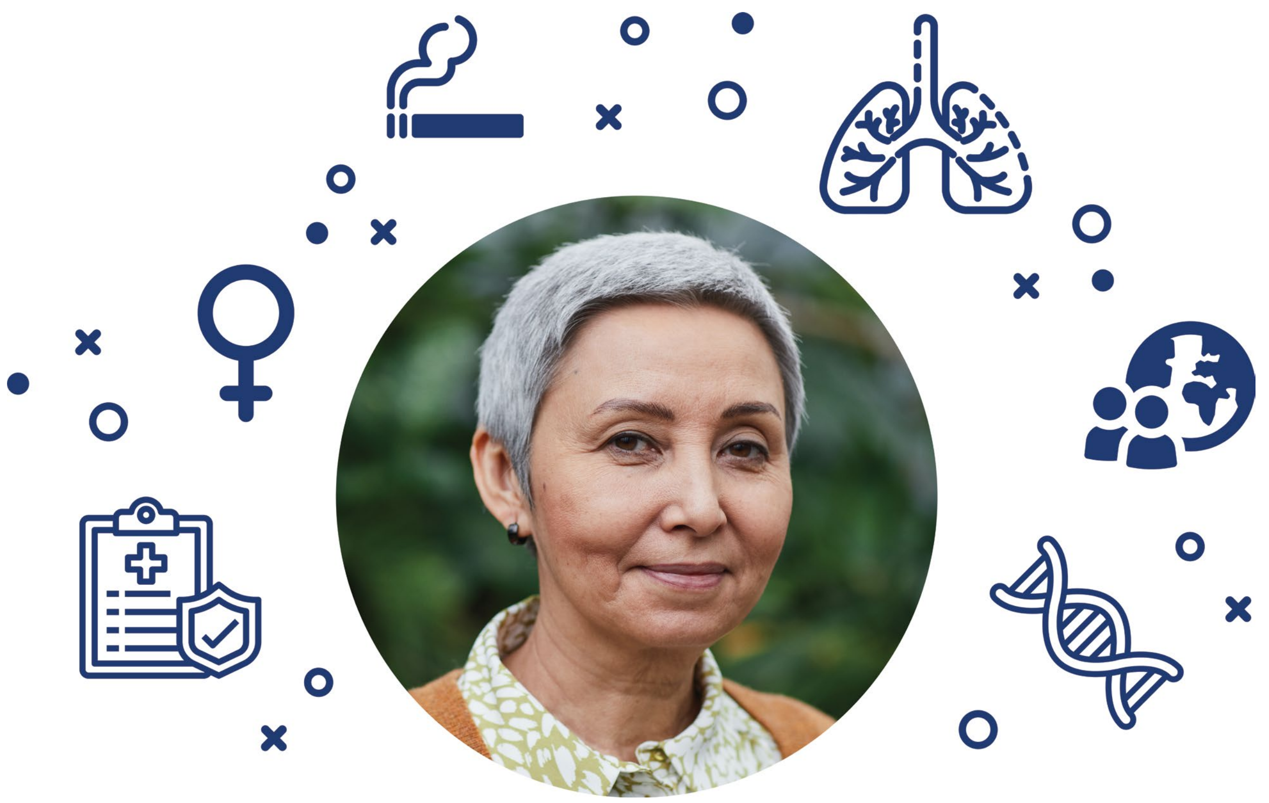

Meet Olivia Tan

- 61-year-old Asian American woman
- Smokes cigarettes
- Family history of lung cancer on her father's side
- Generally healthy
- Gets a chest scan because she smokes and has a family history of lung cancer

AI tools

- AI is used to send Olivia reminders about her chest scan appointment
- Her chest scan is read by an AI tool that predicts whether lung spots are cancer or not
- An app on her smart phone detects poor air quality

Next steps

- Olivia Tan's doctor refers her to a pulmonologist (lung specialist)
- The specialist orders more scans and a biopsy (tissue sample)
- Olivia is advised to stop smoking and gets automated check-up reminders
- She turns on air quality alerts on her phone and wears a mask on poor air quality days

DO AI TOOLS WORK?

Yes — and no. AI tools are powerful and process a lot of data. Sometimes the predictions they make are very accurate but sometimes they don't work as well. One common reason AI tools don't work very well is that the data available to make the tool is incomplete. Let's look at an example:

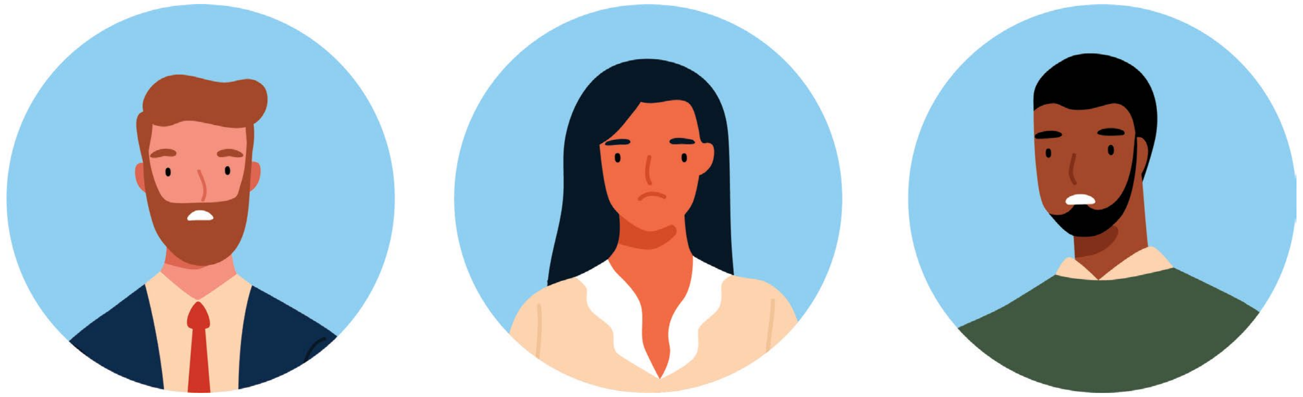

This is Chris, Alicia, and Frederick. They all arrive at their health system on the same day, aren't feeling very well, and have a spot on the back of their hand that wasn't there 6 weeks ago. Their doctors order biopsies and imaging tests as quickly as possible.

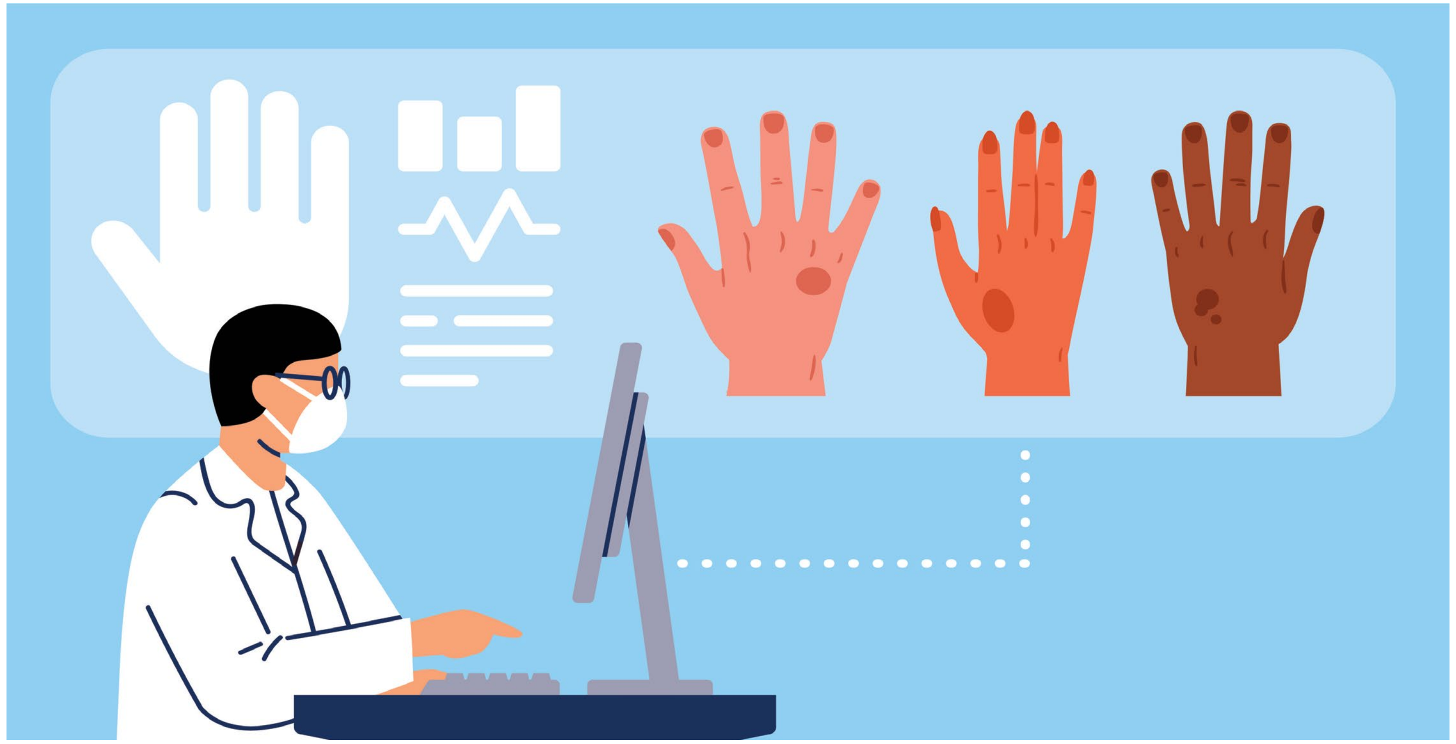

It turns out they all have a similar kind of skin cancer and the same AI tools are used to help diagnose them, but they have different experiences. For Chris, his cancer is detected right away using AI to interpret his imaging results. Alicia's results indicate her cancer might be treatable with a new, but expensive, treatment. But Frederick doesn't get a clear result.

The AI program that detected Chris and Alicia's cancer didn't work for Frederick because it had not been trained with data from enough black patients like Frederick. The AI program gave his provider inconclusive results, so Frederick was told to "just wait and see." His care was delayed, and he didn't get treatment as quickly as Chris and Alicia. Just like with human decision making, there is uncertainty and mistakes can happen when using AI tools.

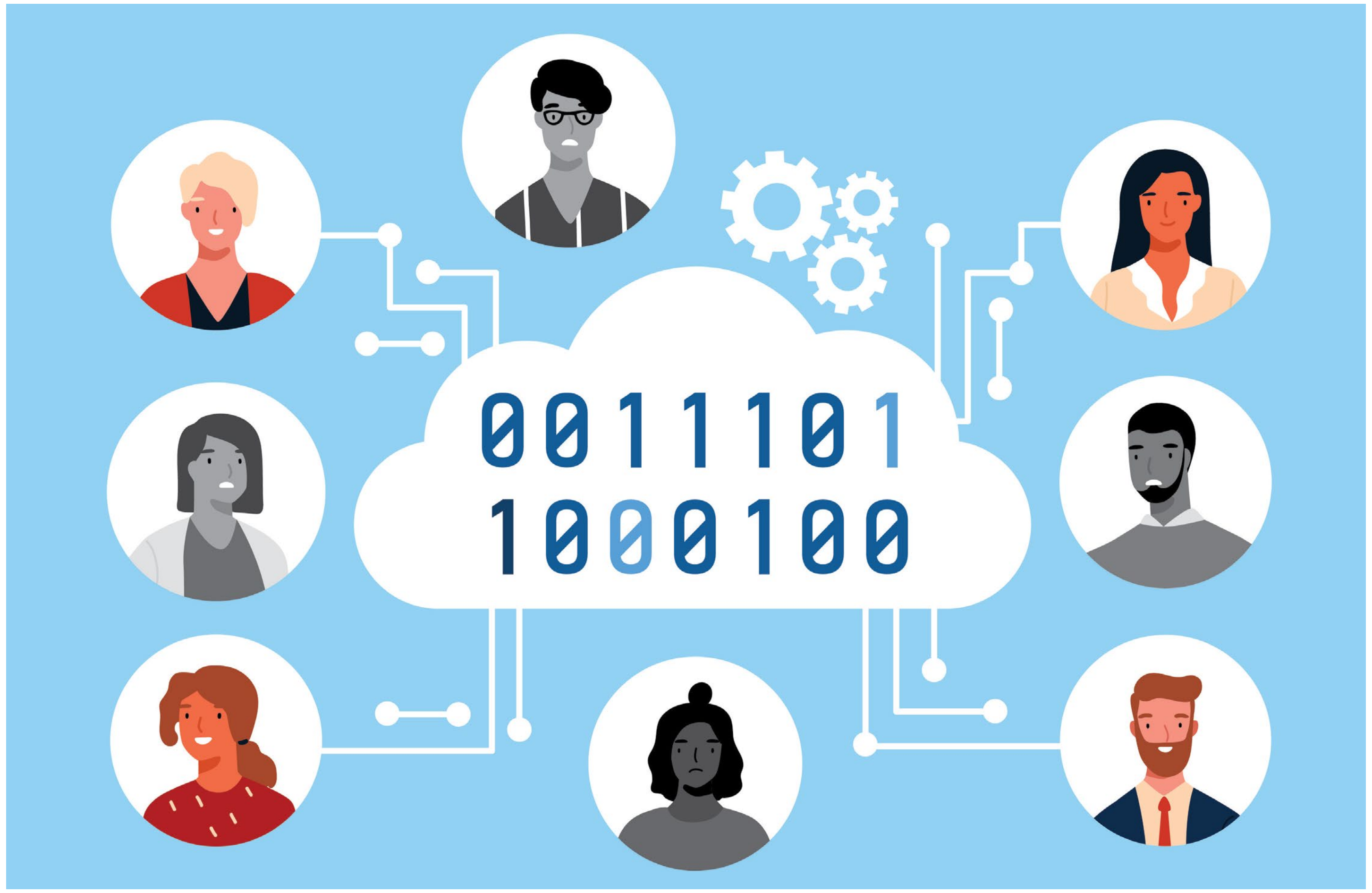

AI improves faster and is more accurate when it includes information about all kinds of people. If some people are left out because they and others like them are not included in the healthcare system or the data used to train a program, AI tools will not work as well for them or the communities they belong to.

WHO USES AI TOOLS IN HEALTHCARE?

Who	How do they use AI?
Healthcare Providers	• Reading X-rays and other images<br>• Diagnosing diseases<br>• Recommending treatments<br>• Predicting health needs of individual patients<br>• Developing personalized treatment plans
Hospitals	• Automated appointment scheduling<br>• Generating discharge summaries<br>• Drafting emails to patients<br>• Billing and insurance claim processing<br>• Predicting future hospital admission
Medical Device Companies	• Insulin pumps<br>• Monitors (heart, oxygen)<br>• Robotic surgical assistants<br>• Medication dispensing
Researchers	• Identifying new treatments and interventions<br>• Understanding the causes of disease
Insurance companies	• Predicting healthcare needs<br>• Predicting future costs
Pharmaceutical Companies	• Drug discovery and development<br>• Clinical trial optimization

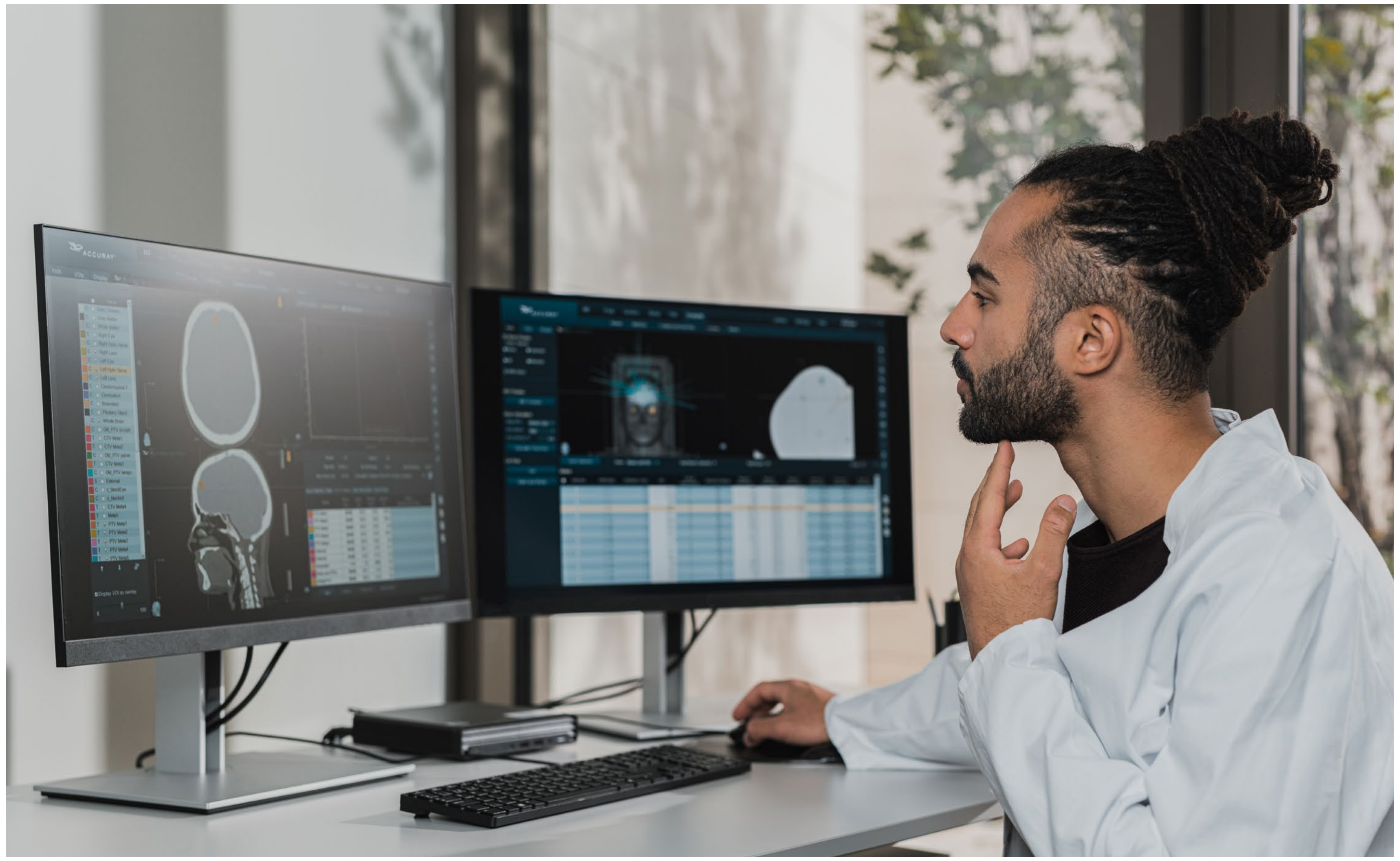

A healthcare provider using AI to assist in reading X-rays and other images.

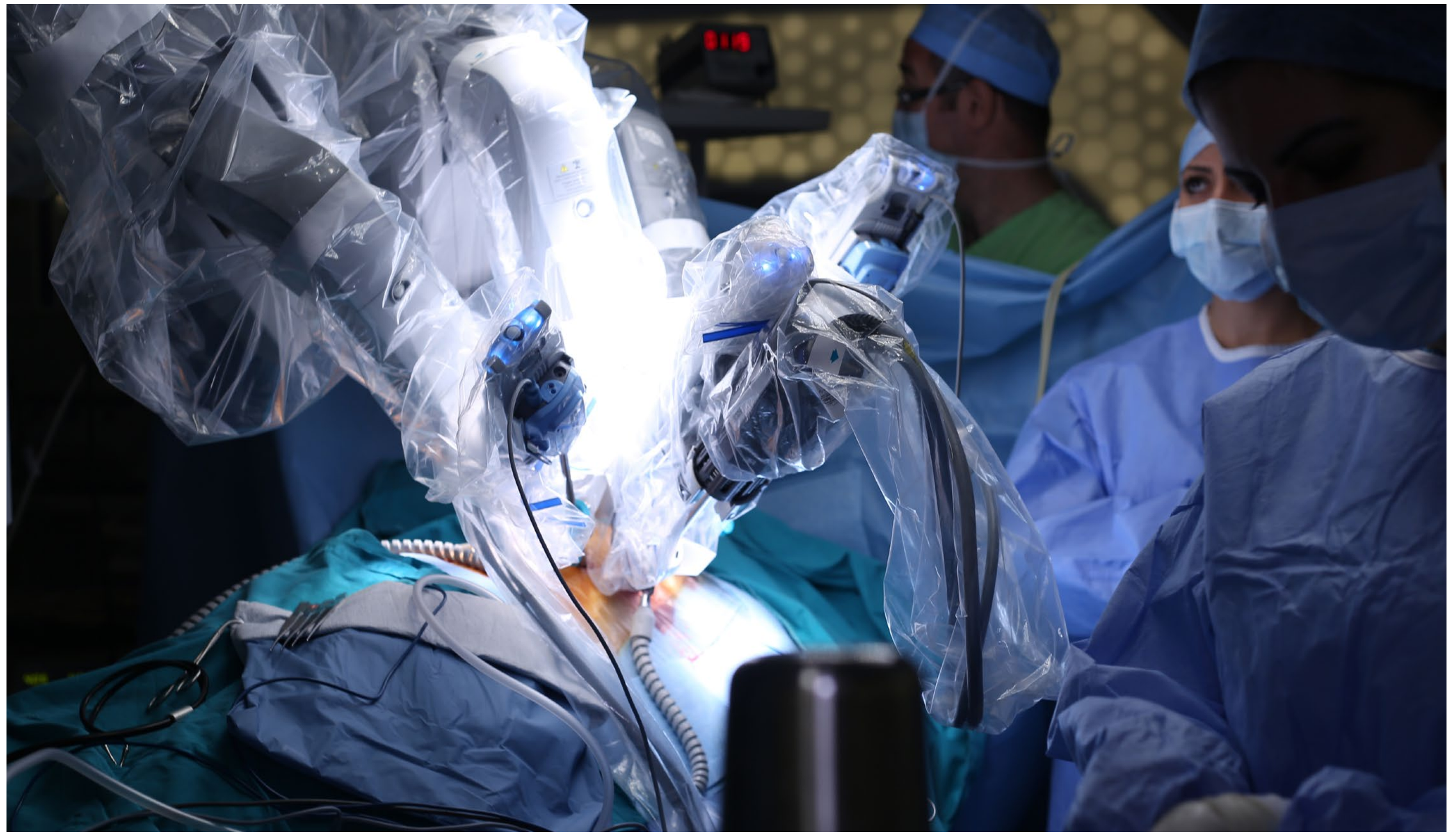

A robotic surgical assistant uses AI to sew stitches.

CAUSE FOR HOPE OR CONCERN?

The use of AI tools in healthcare has both benefits and risks. For example, the large amounts of data used by AI makes AI tools more accurate, but it also raises concerns about privacy. Here we present some of the ethical issues related to the use of AI tools in healthcare.

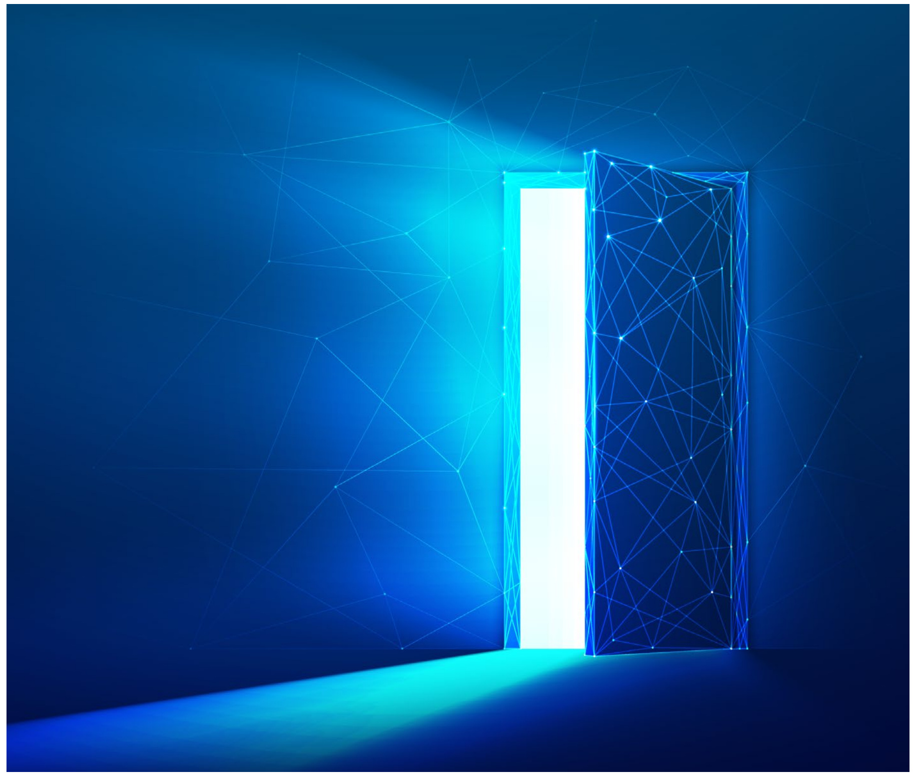

TRUST AND TRANSPARENCY
Patients may not know that AI is being used in healthcare. Patients may want to know where to get assistance if an AI tool doesn't work.

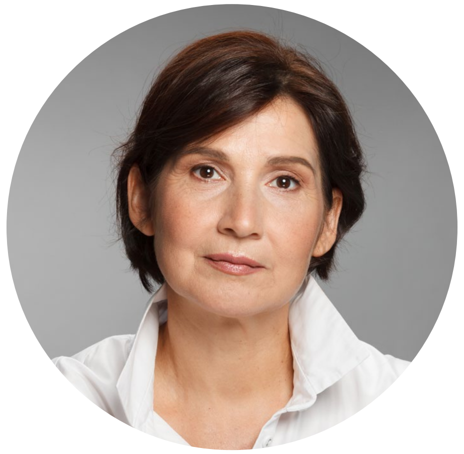

I'd just like to know if AI technology is being used in my healthcare.

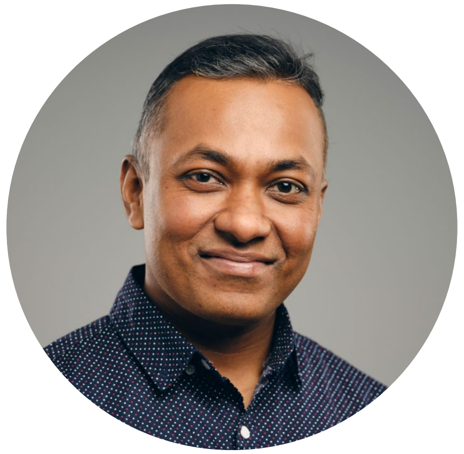

AI is everywhere these days. Why is healthcare any different? I don't really feel I need to know the details.

FAIRNESS
AI tools may not work as well for less represented groups, leading to concerns about bias.

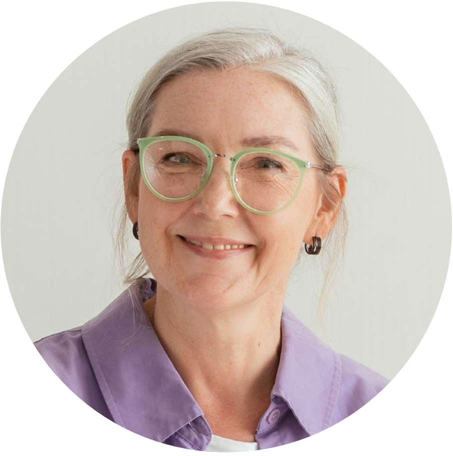

We should use AI tools, even if they don't work for everyone. The benefits far outweigh the risks.

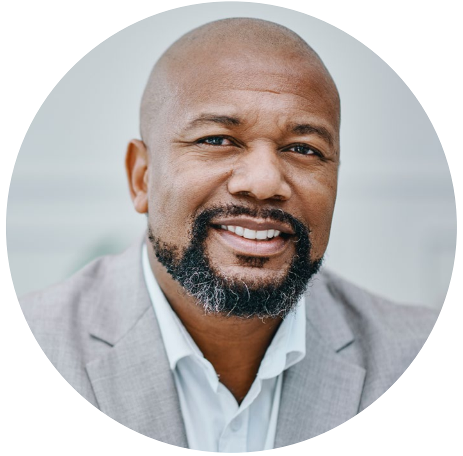

My community is always the last to benefit from these kinds of technologies. Why would I expect AI to be any different?

WILL I STILL BE ABLE TO RELY ON MY DOCTOR?
AI may help doctors do their jobs more effectively. However, doctors and clinics using AI may become too dependent on the technology.

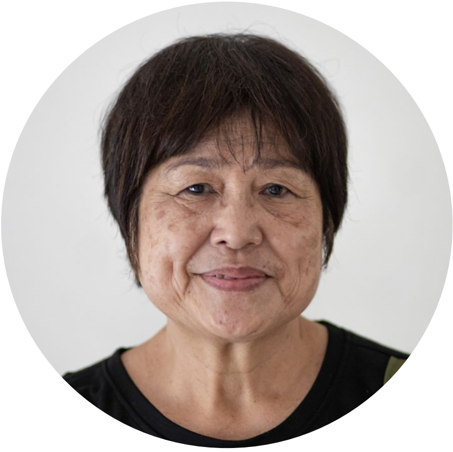

Will my doctor still be making decisions about my care?
Who do I call if there's a problem?

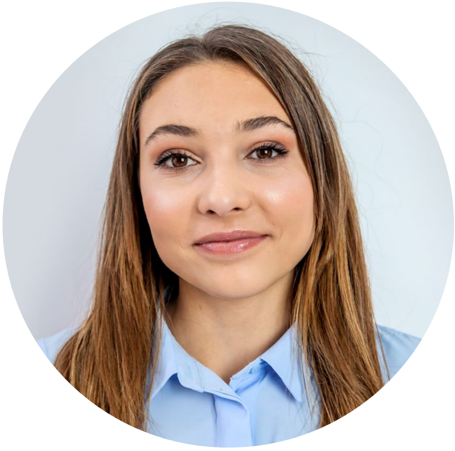

AI can do the work of many people. It will make things easier and a lot more efficient.

WHO BENEFITS?
Using AI technology in healthcare is expensive and not all people or healthcare organizations will have access to high quality tools.

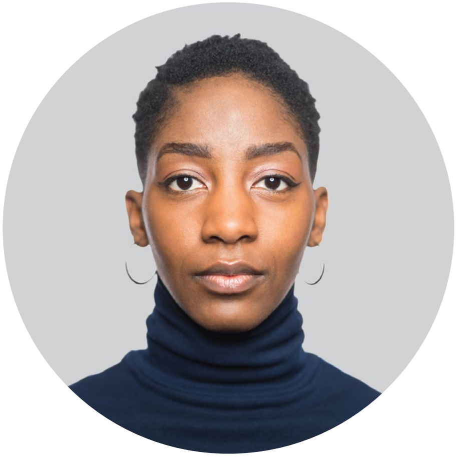

AI is a cool technology, but it will never really benefit me or my community.

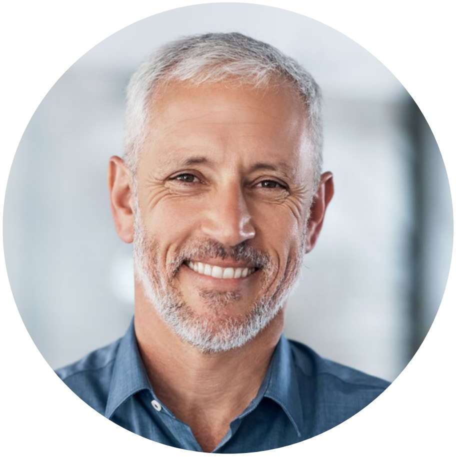

AI tools will be able to help everyone eventually. We need to start somewhere.

OVERSIGHT
As AI is used more in healthcare, there is a need for appropriate oversight to make sure that the technology is used safely, effectively, and ethically.

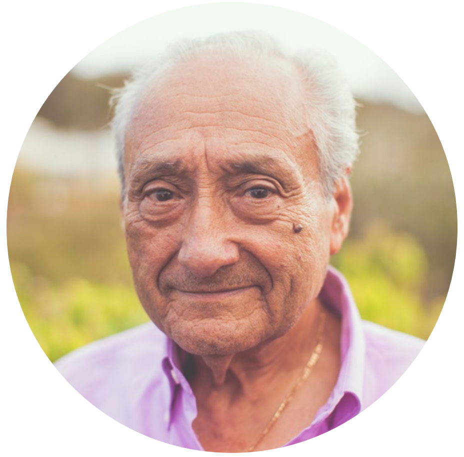

Who is responsible if something goes wrong? If the technology is wrong, is it like malpractice?

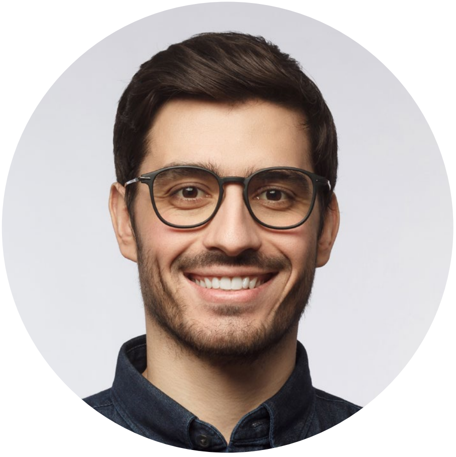

Technology is about innovation. Too many rules will just get in the way.

PRIVACY AND SECURITY
AI needs lots of data to work well. This data often comes from and goes to public agencies, private companies or other organizations outside of the healthcare system. While security measures are taken to safeguard the data, there remains a risk that data can be hacked or systems breakdown.

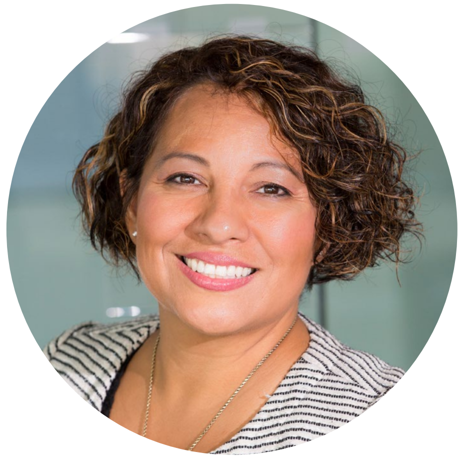

Our data is everywhere. I expect healthcare systems to be careful, but privacy risks are just part of life.

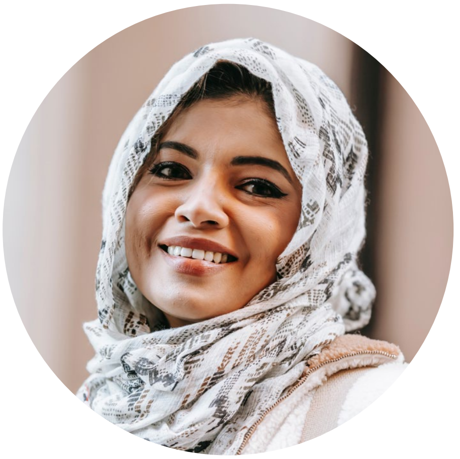

I am not comfortable with my personal information being used to develop AI tools.

WHERE IS THIS HEADED?

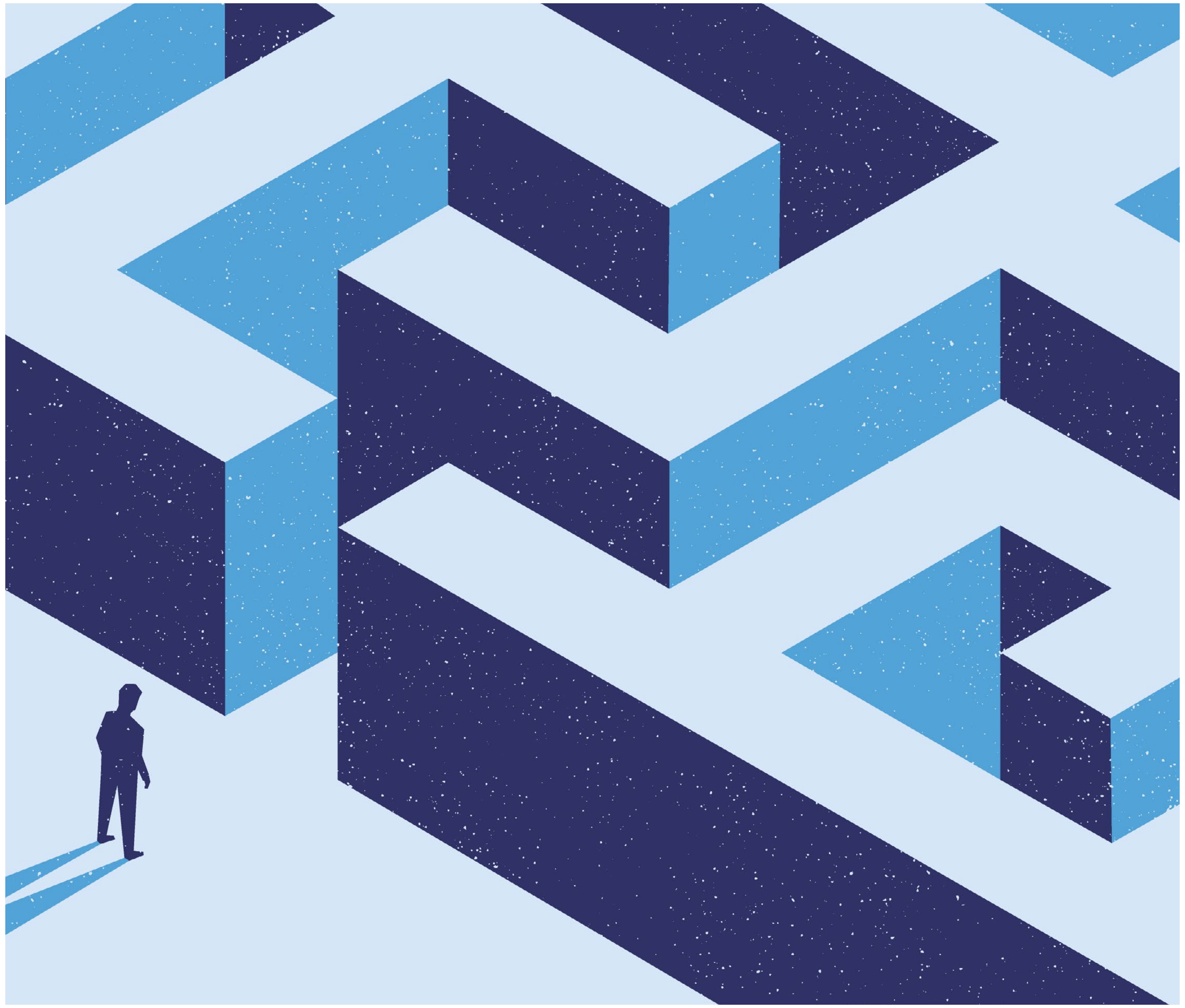

“AI is technology, like any other piece of technology that has benefited us — drugs, cars, planes — AI needs guardrails so we can be protected from the worst failures, while still benefiting from the progress AI offers.”

— March 8, 2023, U.S. Senate hearing

As AI continues to be used in healthcare, new laws and protections are being discussed. These include:

- Requiring companies and health systems to disclose how they use AI
- Holding AI developers to a code of conduct, like the Hippocratic oath (“do no harm”) for physicians
- Mandating testing for safety and effectiveness prior to use
- Embedding warnings or not allowing the use of AI tools trained on incomplete data
- Requiring audits to ensure that the AI tools are safe and effective when used in real-world settings (post-market surveillance)

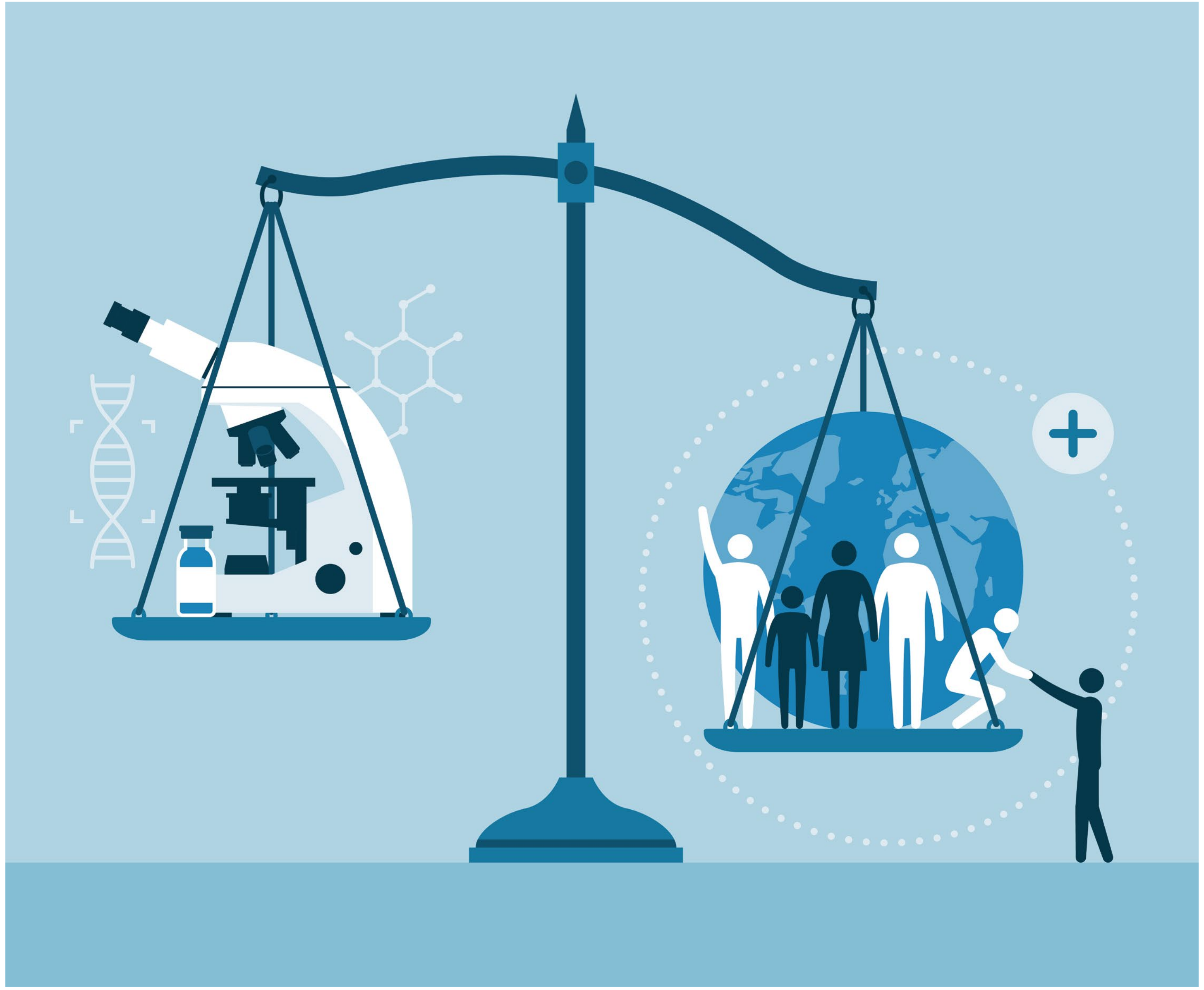

WHAT DOES THE PUBLIC NEED TO KNOW?

In 2022, the White House Office of Science and Technology Policy issued "The Blueprint for an AI Bill of Rights." This document provides guidance on how to protect people when AI is used in healthcare.

One way to tell patients about AI protections is by using labels. For example, nutrition labels on food have been around for decades. They provide information about ingredients, serving sizes, calories, and other nutritional content so people can choose what to buy. Drug labels on medications indicate dosage, expected side effects, refills, and expiration dates.

An AI label could communicate information about what the tool does, the quality of the tool, and how it impacts the patient's care.

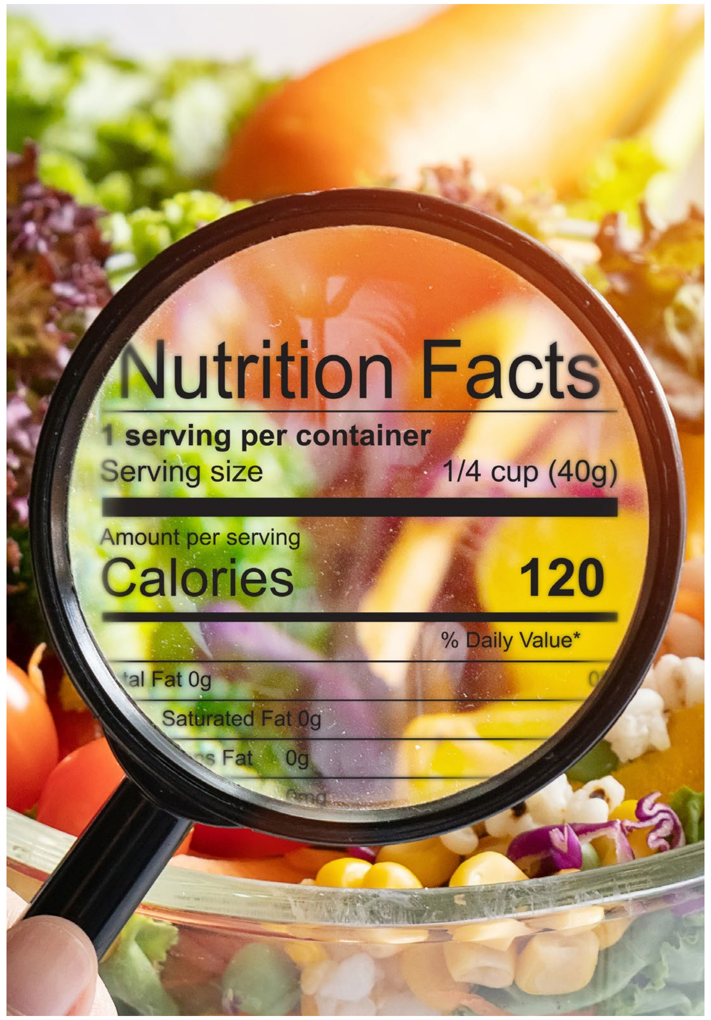

Nutrition label

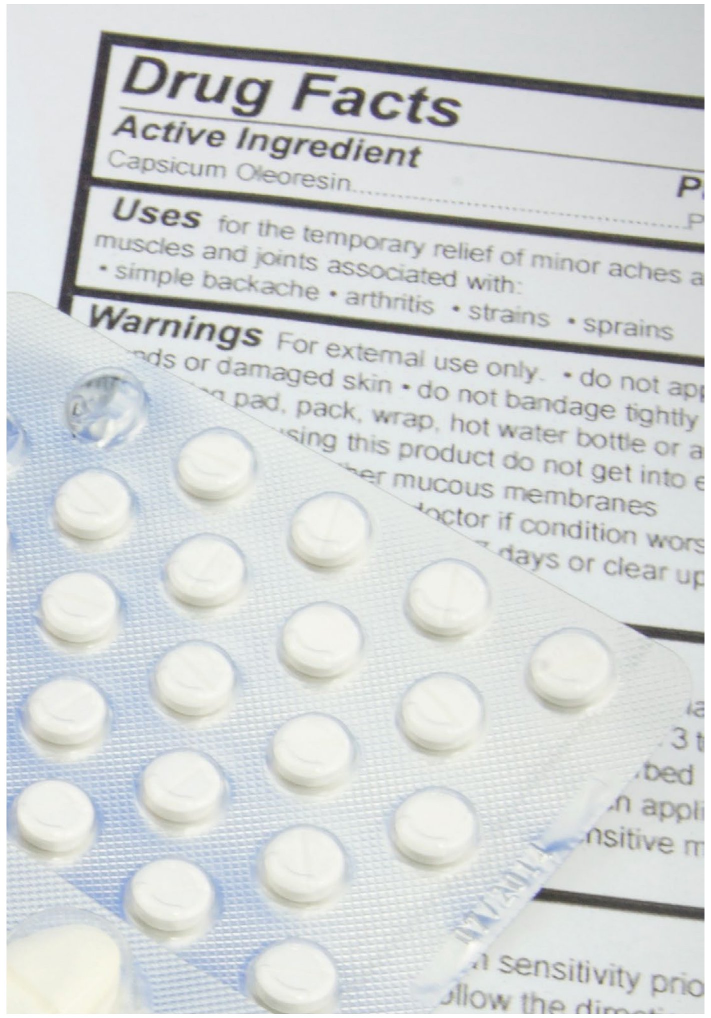

Drug facts

AI that follows the recommendations in the AI Bill of Rights, might include a label that displays information that answers some of the following questions:

- Does the AI tool meet industry standards for safety & effectiveness?
- Can I opt out?
- Does the AI tool improve health?
- Who developed the AI tool?
- Does the AI tool work for all patients regardless of gender, race, ethnicity, age, or disability status?
- Who can I talk to if I have questions?
- How is my privacy is protected?
- Who is responsible for the quality of the AI tool?
- How will I be notified?
- How is the AI tool used in my care?

GLOSSARY OF TERMS

Administrative data
Data collected in the course of providing and/or paying for services (e.g. hospital admissions, physician payment information).

Algorithm
A rule, set of rules, or procedure used for solving a problem or performing a computation.

ChatBot
A chatbot is a computer program that simulates human conversation, either written or spoken, using AI and natural language processing to answer questions and automate routine tasks.

Clinical data
Detailed information about specific aspects of persons, conditions and/or care (e.g. blood pressure, weight, lab results).

Commercial companies
A company that is organized to make a profit.

Commercialization
The sharing of health information for the purpose of making money.

De-identified data
Data where the personal identifiers of the individuals have been removed.

Electronic health record (EHR) or electronic medical record (EMR)
Health histories collected from patient visits to clinics and hospitals that can be shared electronically.

Individual-level health data
Data that are collected from individuals and that can be associated with an individual such as medical records, blood test results, exercise habits, disease risk, and prescription medicines.

Health Data
Any type of information that contains information about the health conditions of a person or group of people. “Health data” might include biospecimens (e.g., blood/tissue samples).

Health provider
A person or company that takes care of patients.

Healthcare system
An organized group of people, institutions, and resources that deliver healthcare services to meet the health needs of populations.

Informed consent
A contract agreement that is meant to ensure that research subjects and patients have made a voluntary decision to participate in research after its purpose, risks and benefits have been fully explained.

Natural Language Processing
A field of artificial intelligence that focuses on the interaction between computers and human language, enabling machines to understand, interpret, and generate human language.

Notification
The alerting of patients to uses of their health data so that they’re aware of it.

Patient Portal
A digital file of individual health data on a technology device that can be used and shared. Patients can access their health data such as recent doctor visits, medications, and lab results through the patient portal.

Private company
Any privately owned business, corporation, or enterprise.

GLOSSARY OF TERMS CONT.

Public deliberation
A community discussion that can inform policy decisions on issues that affect members of the public.

Research
The systematic investigation into and study of materials and sources to establish facts and reach new, generalizable conclusions.

Survey data
Information collected directly from and about individuals or groups.

Tracking data
Data that exists about individuals in the context of everyday life (e.g. Fitbit, web activity, GPS tracking).

CREDITS

This booklet was prepared by the AI in Healthcare research team at the University of Michigan

Gloria Carmona
Allen Flynn
Charles Friedman
Adnan Hammad
Reema Hamasha
Sharon Kardia
Charo Ledon
Blackford Middleton
Paige Nong
Jodyn Platt
Joshua Richardson
Veronica Romanov
Kerry Ryan
Morgan Sielaff
Renee Smiddy
Abram Wagner

This project, “Public Trust of Artificial Intelligence in the Precision CDS Health Ecosystem” is funded by a grant from the National Institutes of Health, National Institute of Biomedical Imaging and Bioengineering (5R01EB030492).

The Use Of Artificial Intelligence And Smart Technology Tools In Health Care: A Public Deliberation© 2024 by Jodyn Platt and the AI in Healthcare research team is licensed under CC BY-NC-SA 4.0. To view a copy of this license, visit: creativecommons.org/licenses/by-nc-sa/4.0

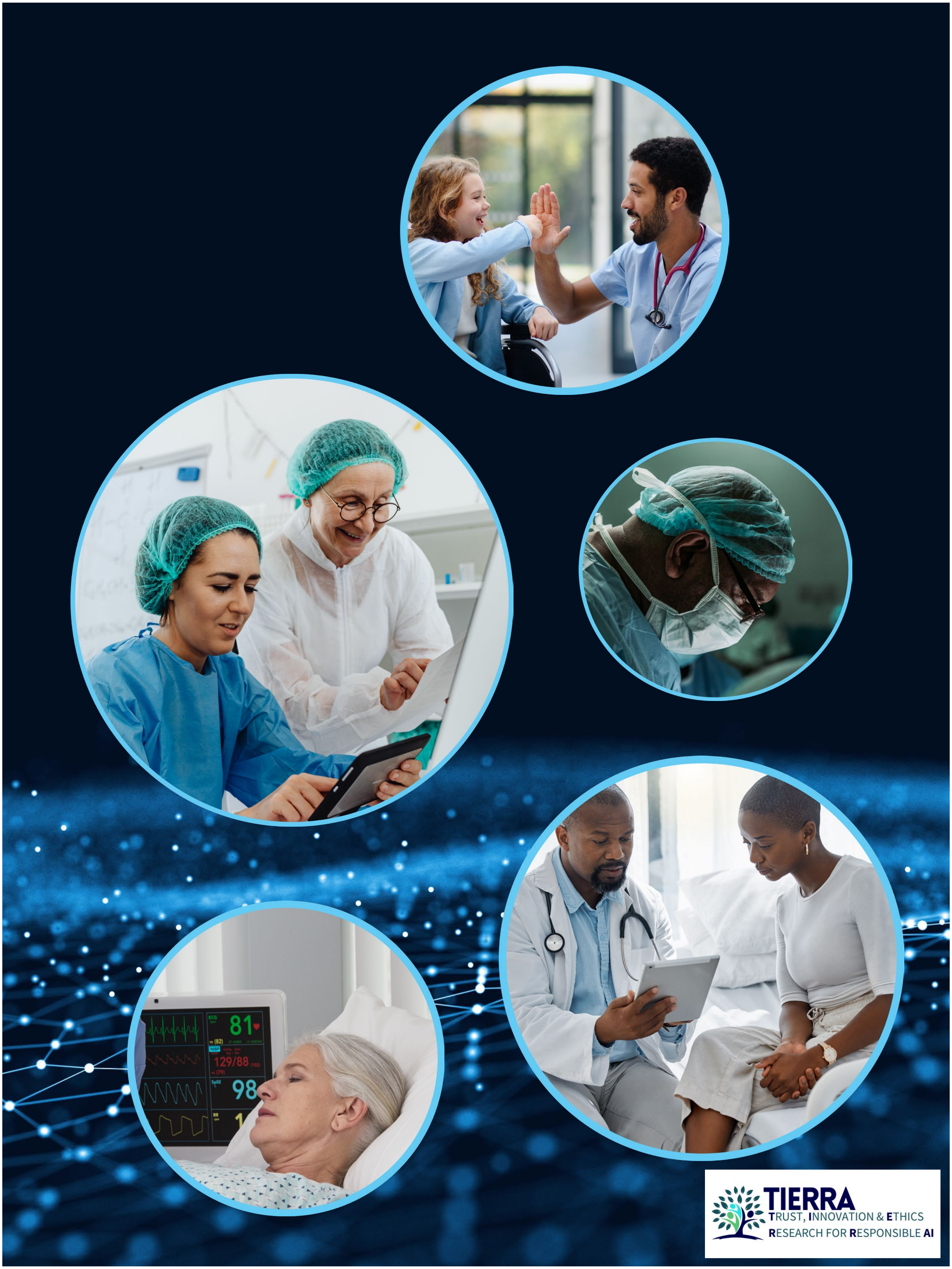
81
129/88
98
TIERRA
TRUST, INNOVATION & ETHICS
RESEARCH FOR RESPONSIBLE AI

AI in Healthcare Presentation

AI in Healthcare

A Deliberative Democracy Session

UNIVERSITY OF MICHIGAN

HUM00240942

5-R01-EB030492-03

1

WELCOME

We'll get started at 9:10 am

UNIVERSITY OF MICHIGAN

2

Reminders

- This event is being Zoom recorded.
  - Recordings will be used to make transcripts
  - We will remove names and identifying information from the transcripts
- Please have the packet you received in the mail with you for this meeting
  - You can open the slide packet now, if you have not already done so.

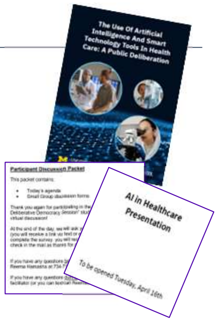

3

INTRODUCTIONS

Who's here?

UNIVERSITY OF MICHIGAN

4

Our Study

- The use of Artificial Intelligence in healthcare is increasing.
  - Lots of data from everyone
  - Lots of different types of people and organizations needed to make it happen
- The goal of this study is to understand public perspectives on the use of AI in heath care.

Our study is supported by the National Institutes of Health (NIH) National Institute of Biomedical Imaging and Bioengineering (5-R01-EB030492-03)

5

Deliberation Overview

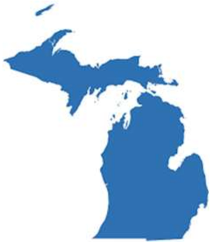

- Today's session is one of five deliberations that are being held in Michigan in 2024.
- We want to hear from different communities around the state.

6

Our Community Partners

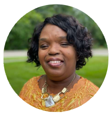

Susie Williamson

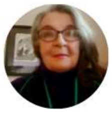

Veronica Romanov

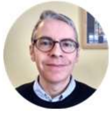

Owen Goslin

7

What is a public deliberation?

A **public deliberation** is a community discussion that brings community members into the process of making decisions or solving problems that affect them. Public deliberations can be in-person or they can be virtual meetings.

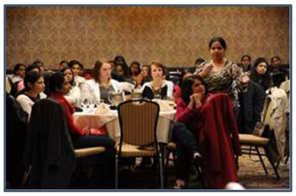

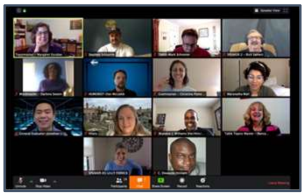

8

Key questions

- What are your hopes and concerns about the use of AI in healthcare?
- What information does the public need about the use of AI in healthcare?

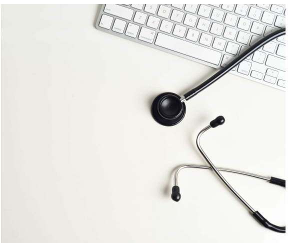

9

Ground Rules

- Be respectful. Listen well.
- Keep an open mind.
- Only 1 person talk at a time. Try not to interrupt.
- Give reasons for your opinions.
- You're not required or expected to share your personal information.
- If others share personal information, please don't share it outside today's session.

10

Video Calling Tips

Find a quiet place and turn off phone notifications.

For best results, close other apps before starting Zoom call.

Turn on video!

Mute yourself when you aren't speaking.

Audio or video freezing? You may have a bad internet connection. Try turning your video on and off.

Wear headphones if you have them.

Be respectful!

11

Today's Agenda

9:00 - 9:30	Welcome and introductions
9:30 - 10:15	Session 1 presentation: AI in Healthcare
10:15 - 10:25	Session 1 Q&A
10:25 - 10:30	5-minute break
10:30 - 11:30	Breakout Room discussion: Icebreaker & Perspectives on AI
11:30 - NOON	Break for Lunch
12:00 - 12:45	Session 2 presentation and Q&A: AI Policy
12:45 - 1:45	Breakout Room discussion: AI Tool Label Activity
1:45 - 1:50	5-minute break
1:50 - 2:30	Large group discussion & review

12

SESSION 1

AI in Healthcare

UNIVERSITY OF MICHIGAN

13

Session 1 Presentations

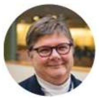

SHARON KARDIA
Public health

Part 1: What is AI?

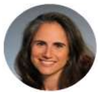

JODYN PLATT
Learning Health Systems

Part 2: Ethical considerations

14

Discussion Questions

- What are the risks and benefits of using AI in healthcare?
  - What about for the care of your family, friends, or community?
- How is AI in healthcare the same or different from how AI is already used in society (GPS, Siri/ Alexa, Facial Recognition)?

15

What is AI?

- Uses a lot data
- Detects patterns
- Predicts future events based on past patterns
- Example: Google Maps and predicted arrival times

16

How is AI used in society today?

AI in Society
Siri, Alexa
GPS navigation in cars and phones
Targeted coupons
Banking fraud alerts
Netflix/ Amazon recommendations
Stock market trends
Superbowl winner
Facial recognition software
Transcription (voice recognition)
Handwriting to text
Weather forecasting

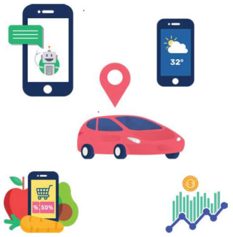

17

This is happening in medicine, too

AI in Society	AI in Healthcare
Siri, Alexa	Home health reminders
GPS navigation in cars and phones	Ambulance routing
Targeted coupons	Pharmaceutical recommendations
Banking fraud alerts	Sepsis alerts
Netflix/ Amazon recommendations	Treatment plans
Stock market trends	Disease risk
Superbowl winner	Surgical risk assessment
Facial recognition software	Radiology/ image interpretation
Transcription (voice recognition)	Medical notes
Handwriting to text	Test orders
Weather forecasting	Prognosis

18

What is happening in radiology right now

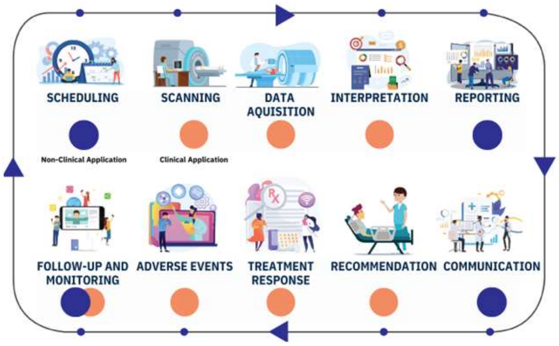

19

Who uses AI Tools in Healthcare?

Healthcare Providers	Hospitals	Medical Device Companies	Researchers	Insurance companies	Pharmaceutical Companies
• Reading X-rays and other images<br>• Diagnosing diseases<br>• Recommending treatments<br>• Developing personalized treatment plans	• Automated appointment scheduling<br>• Generating discharge summaries<br>• Drafting emails to patients<br>• Billing and insurance claim processing<br>• Predicting future hospital admission	• Insulin pumps<br>• Oxygen monitors<br>• Heart monitors<br>• Robotic surgical assistants<br>• Medication dispensing	• Identifying new treatments and interventions<br>• Understanding the causes of disease	• Predicting health needs<br>• Predicting future costs	• Drug discovery and development<br>• Clinical trial optimization

20

What Kind of Data do AI Tools Use in Healthcare?

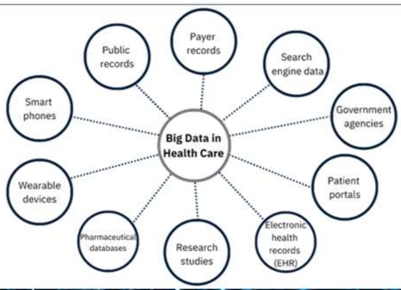

21

What Kind of Data do AI Tools Use in Healthcare?

- Lifestyle / behavior
  - Diet and nutrition
- Demographics
  - Sex, age, race
- Medical History
  - Height, weight
  - Family history of cancer
- Social Determinants of Health
  - Zip code, food insecurity

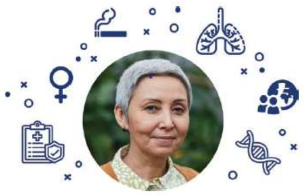

22

Is the doctor involved?

	Assistive AI algorithms		Autonomous AI algorithms		
	Level 1	Level 2	Level 3	Level 4	Level 5
**Example**	AI analyzes mammogram and indicates high risk regions	AI analyzes mammogram and provides a risk score	AI analyzes mammogram and recommends biopsy, with clinician back up	AI analyzes mammogram and recommends biopsy, *without* clinician back up	AI analyzes mammogram and recommends biopsy, *without* clinician back up<br><br>Applied system-wide
**Who responds to AI output**	Clinician	Clinician and AI	AI	AI	AI
**Who's checking the outcome**	Clinician/ Health system	Clinician	AI, with Clinician as backup	AI	AI

ADAPTED FROM: Bitterman DS, Aerts HJ, Mak RH. Approaching autonomy in medical artificial intelligence. The Lancet Digital Health. 2020 Sep 1;2(9):e447-9.

23

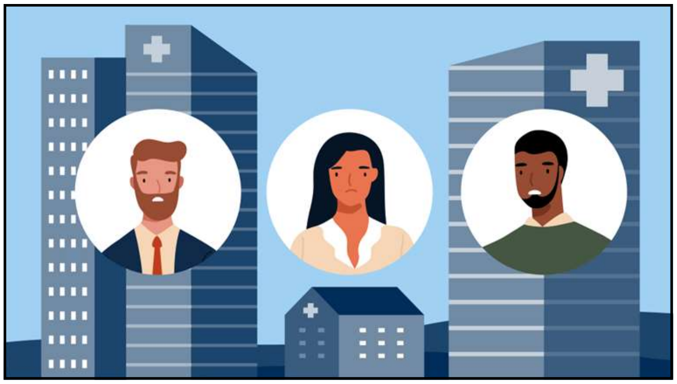

24

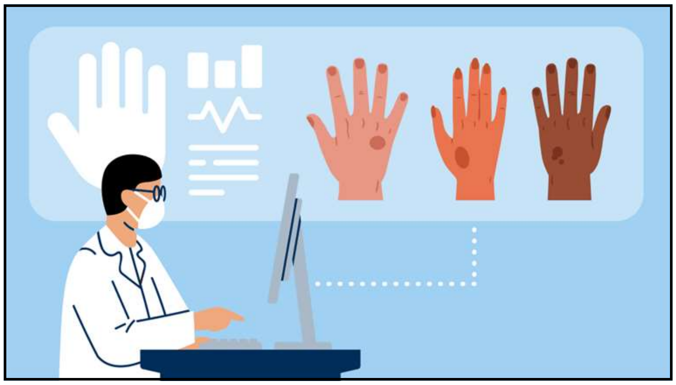

25

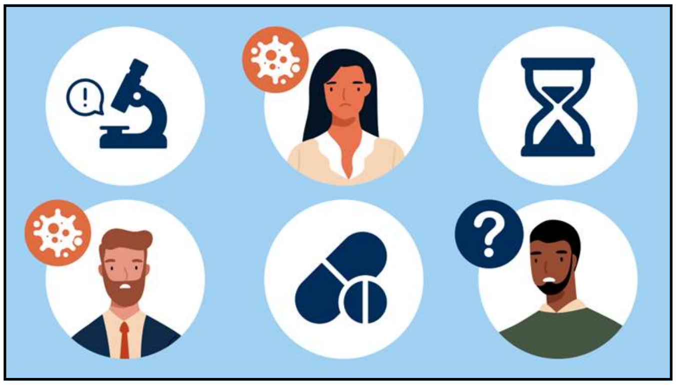

26

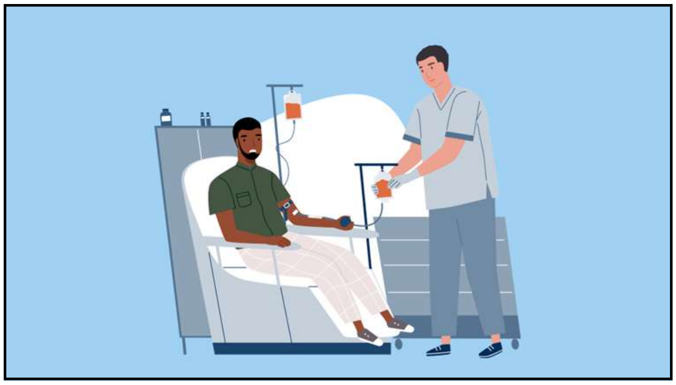

27

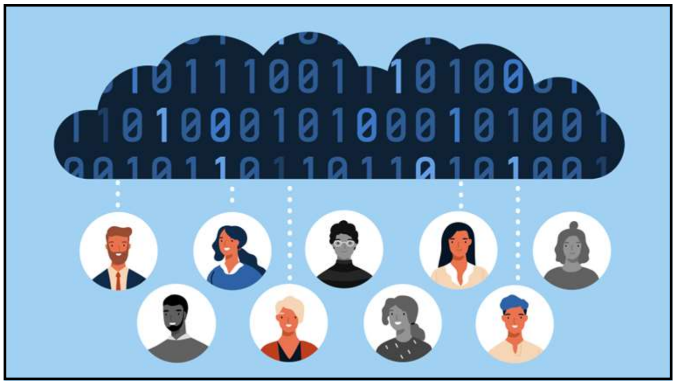

28

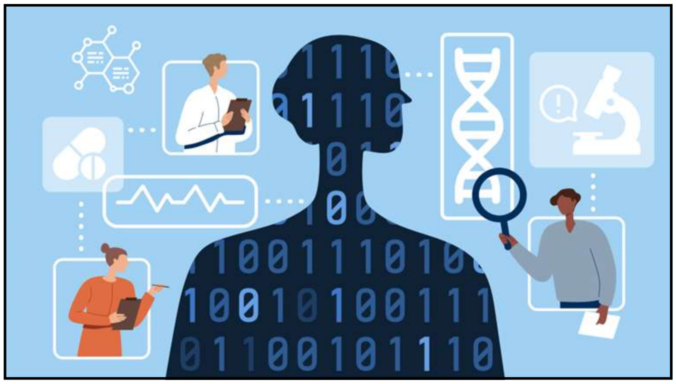

29

Conclusion

- AI tools use a lot of data
  - Identify trends to predict the future
- Broad range of applications
- Do AI tools work?
  - Often they do. Sometimes they don't. It depends.

30

Session 1 Presentations

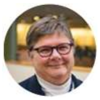

SHARON KARDIA
Public health

Part 1: What is AI?

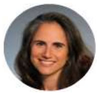

JODYN PLATT
Learning Health Systems

Part 2: Ethical considerations

31

Discussion Questions

- What are the risks and benefits of using AI in healthcare?
  - What about for the care of your family, friends, or community?
- How is AI in healthcare the same or different from how AI is already used in society (GPS, Siri/ Alexa, Facial Recognition)?

32

Ethical issues

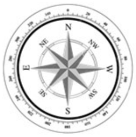

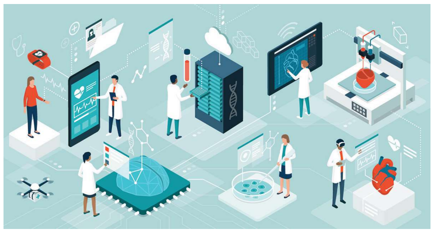

33

Examples of ethical issues

- Fairness
- Relationships between people
- Privacy and security
- Regulation and oversight

34

Fairness

- Does AI need to treat everyone the same?
- Should we use AI to address inequity?

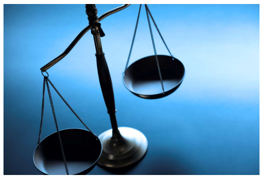

35

Fairness

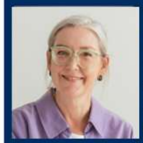

"We should use AI tools, even if they don't work for everyone. The benefits far outweigh the risks."

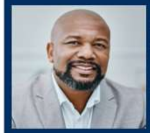

"My community is always the last to benefit from these kinds of technologies. Why would I expect AI to be any different?"

36

Relationships between people

- Will AI make it easier to get answers to your questions? Harder?
- Will it change how you talk to your doctor or how your doctor talks to you?

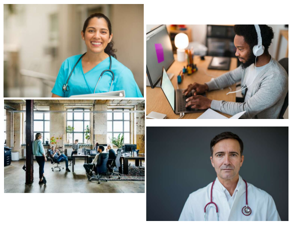

37

Relationships between people

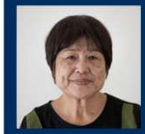

"Will my doctor still be making decisions about my care? Who do I call if there's a problem?"

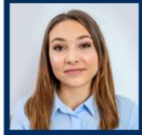

"AI can do the work of many people. It will make things easier and a lot more efficient."

38

Privacy and security

- "Big data" = Volume, Velocity, Veracity (quality), Variety
- Open system
- How are individuals protected?

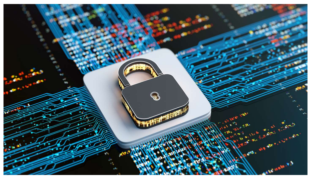

39

Privacy and Security

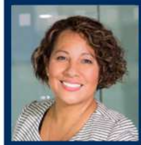

"Our data is everywhere. I expect healthcare systems to be careful, but privacy risks are just a part of life these days."

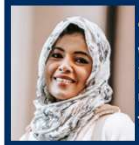

"I am not comfortable with my personal information being used to develop AI tools."

40

Regulation and Oversight

- Developers
- Companies
- Clinicians
- Health system
- Regulators
- No one?

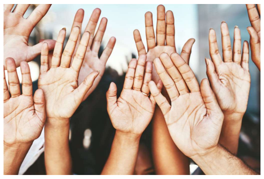

41

Regulation and Oversight

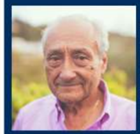

"Is anyone responsible if something goes wrong? I know about malpractice, but what if the technology is wrong?"

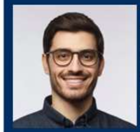

"Technology is about innovation. Too many rules will just get in the way."

42

Discussion Questions

- What are the risks and benefits of using AI in healthcare?
  - What about for the care of your family, friends, or community?
- How is AI in healthcare the same or different from how AI is already used in society (GPS, Siri/ Alexa, Facial Recognition)?

43

Q&A

Questions for the presenters

UNIVERSITY OF MICHIGAN

44

Today's Agenda

9:00 - 9:30	Welcome and introductions
9:30 - 10:15	Session 1 presentation: AI in Healthcare
10:15 - 10:25	Session 1 Q&A
10:25 - 10:30	5-minute break
10:30 - 11:30	Breakout Room discussion: Icebreaker & Perspectives on AI
11:30 - NOON	Break for Lunch
12:00 - 12:45	Session 2 presentation and Q&A: AI Policy
12:45 - 1:45	Breakout Room discussion: AI Tool Label Activity
1:45 - 1:50	5-minute break
1:50 - 2:30	Large group discussion & review

45

Breakout Room Discussion

Icebreaker & Perspectives on AI

UNIVERSITY OF MICHIGAN

46

Breakout Room Discussion Overview

- Quick Icebreaker
- Perspectives on AI
  - What stood out to you?
  - What do you see as the risks and benefits?
  - What are you concerned about? What are you excited about?
  - What information do you want to know?

47

LUNCH BREAK

Please log back in at Noon!

UNIVERSITY OF MICHIGAN

48

WELCOME BACK!

We'll get started at 12:05 pm

UNIVERSITY OF MICHIGAN

49

Today's Agenda

9:00 - 9:30	Welcome and introductions
9:30 - 10:15	Session 1 presentation: AI in Healthcare
10:15 - 10:25	Session 1 Q&A
10:25 - 10:30	5-minute break
10:30 - 11:30	Breakout Room discussion: Icebreaker & Perspectives on AI
**11:30 - NOON**	**Break for Lunch**
12:00 - 12:45	Session 2 presentation and Q&A: AI Policy
12:45 - 1:45	Breakout Room discussion: AI Tool Label Activity
1:45 - 1:50	5-minute break
1:50 - 2:30	Large group discussion & review

50

SESSION 2

AI Policy: How do we tell people this is happening?

UNIVERSITY OF MICHIGAN

51

Session 2 Presentations

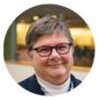

SHARON KARDIA
Public health

Part 1: AI Policy & Labeling

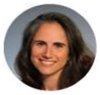

JODYN PLATT
Learning Health Systems

Part 2: Sample Voting and AI Tool Labels

52

How we could decide "do" or "don't"

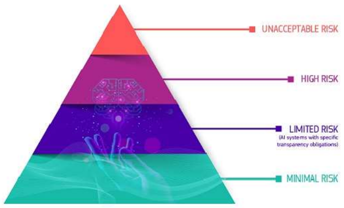

EU Proposed regulatory framework

https://digital-strategy.ec.europa.eu/en/policies/regulatory-framework-ai

53

How we could decide "do" or "don't"

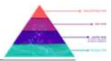

Risk level*	Examples	Policy
Unacceptable	Criminal activity	Prohibit all use
High	Robotic surgery; Diagnosis	Require notification; Review and monitor
Limited	Chatbot used for customer service	Require notification; offer alternatives
Minimal	Netflix recommendations	No restrictions

54

How we could decide ”do” or “don’t”

* Risk to People? Society? Doctors? Health systems? Companies?

Risk level*	Examples	Policy
Unacceptable	Criminal activity	Prohibit all use
High	Robotic surgery: Diagnosis	Require notification; Review and monitor
Limited	Chatbot used for customer service	Require notification; offer alternatives
Minimal	Netflix recommendations	No restrictions

55

In this deliberation we focus on…

Risk level*	Examples	Policy
Unacceptable	Criminal activity	Prohibit all use
High	Robotic surgery; Diagnosis	Require notification; Review and monitor
Limited	Chatbot used for customer service	Require notification; offer alternatives
Minimal	Netflix recommendations	No restrictions

Our focus today

56

Other examples of “limited risk”

- Chat bots for customer service
  - Scheduling appointments
  - Recommending routine screening
- Emails from your doctor, written or drafted by AI
- Clinic notes or summaries, written or drafted by AI

57

Other examples of “high risk”

- AI-generated treatment plan
- Organ transplant decision-making
- Monitoring of vitals (sepsis, blood oxygen)
- Clinical trials of new treatments

Most clinical applications are classified as high risk.

58

Discussion Question

- What needs to be included on a “label” that would notify people that AI is being used in healthcare?

59

Labels as notification

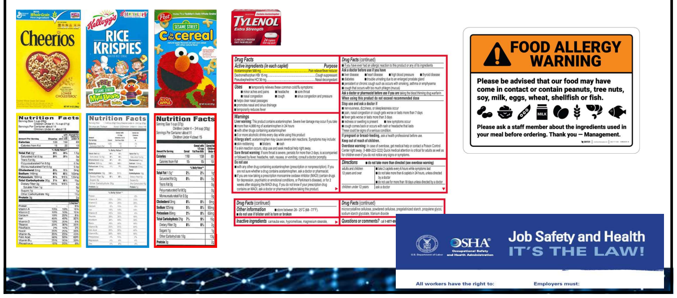

60

How could you be notified about AI?

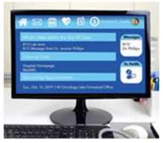

Posted in patient portals

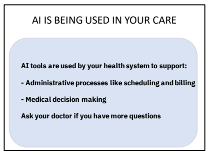

Posted in your clinic

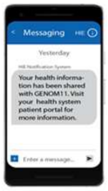

Smart phone

61

Policies relevant to AI in Healthcare

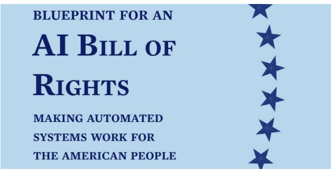

- Safe and effective systems
- Algorithmic discrimination protections
- Data privacy
- Notice and explanation
- Access to people when you need help

62

Policies relevant to AI in Healthcare

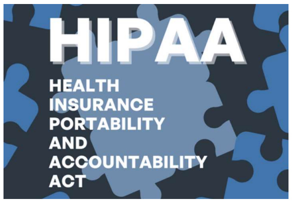

- Sets rules for *data* use, sharing, & security
  - Considers purpose and user of data to set rules about sharing, security, privacy
- HIPAA rules apply to data used by AI

63

Policies relevant to AI in Healthcare

- Approves medical devices and drugs as "safe and effective"
- Enforces safety & reviews performance
- Some AI tools considered "devices"
- Many health AI tools are not regulated by FDA

64

Goal: Trust and Transparency

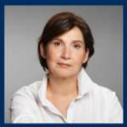

"I'd just like to know if AI technology is being used in my healthcare."

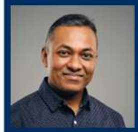

"AI is everywhere these days. Why is healthcare any different? I don't really feel like I need to know."

65

Session 2 Presentations

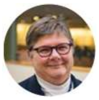

SHARON KARDIA
Public health

Part 1: AI Policy & Labeling

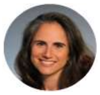

JODYN PLATT
Learning Health Systems

Part 2: AI Tool Labels

66

Discussion Questions

What needs to be included on a "label" that would notify people that AI is being used in healthcare?

- What is useful to know about an AI tool that is used in your healthcare?
- What questions do you want answered about an AI tool?
- What is more or less important to know?

67

Anatomy of a label

- Standardized (familiar) format
- Designed to highlight important information
- Comprehensive to provide complete information
- **Goal:** To provide consumers the information they need to make informed decisions about whether to use a product

68

What you've told us so far

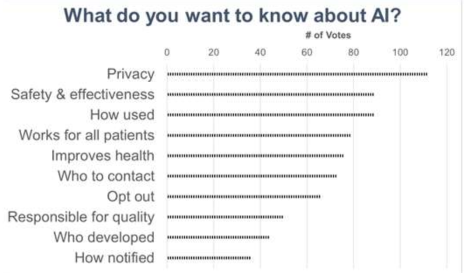

69

AI Label Activity

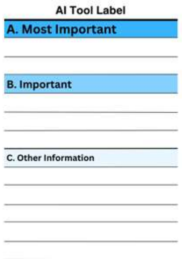

What needs to be included on a "label" that would notify people that AI is being used in healthcare?

70

Information on an AI tool label

Who developed the AI tool?

Company
Health system ("home grown")

71

Information on an AI tool label

Does it meet standards of safety and effectiveness?

Does the AI tool comply with the FDA?
Has it proved to be effective in clinical trials?
Has it passed safety tests?

Who is responsible for the quality of the AI tool?

Developer?
Health system?
End user? (Clinician, patient)

72

Information on an AI tool label

How will I be notified when the AI tool is used in my healthcare?

- Will I be contacted via my patient portal?
- Will it be documented in my medical records?
- Will my doctor tell me about it?

How is the AI tool used in my healthcare?

- What role does it play in my healthcare?
- How does it use my health information?
- Does the AI tool make decisions or does my doctor?

73

AI TOOL LABEL QUESTIONS

Who do I talk if I have questions about the AI tool?

- Is there a website with more information?
- Is there a contact person?
- Should I ask my doctor?

Can I opt out of having an AI tool used in my healthcare?

- Can I have a human do this task instead?
- What happens if I opt out?
- What are my options?

74

Information on an AI tool label

How does the AI tool improve health for patients?

- Does the tool reduce the time to diagnosis or treatment?
- Does it improve accuracy of results?
- Does it improve management of my care?

Does the AI tool work for all patients regardless of gender, race, ethnicity, age, or disability status?

- Does it work for everyone equally?
- Has it been tested on diverse patient groups?
- How does it avoid bias?

75

AI TOOL LABEL QUESTIONS

How is my privacy protected?

- Who has access to my data?
- Is my data encrypted?
- Is my data de-identified?
- What safeguards are in place?

76

Anything else?

?

77

Your Task

AI Tool Label

A. Most Important

B. Important

C. Other Information

For the AI tool, I want to know...

- who is **responsible for the quality** of the AI tool.
- **who developed** the AI tool.
- **who to contact** if I have questions.
- how I will be **notified** when the AI tool is used.
- how the AI tool is **used** in my care.
- how my **privacy** is protected.
- if I can **opt out**.
- if the AI tool **improves health**.
- if the AI tool **works for all patients** regardless of gender, race, ethnicity, age, or disability status.
- if the AI tool meets industry standards for **safety and effectiveness**.

78

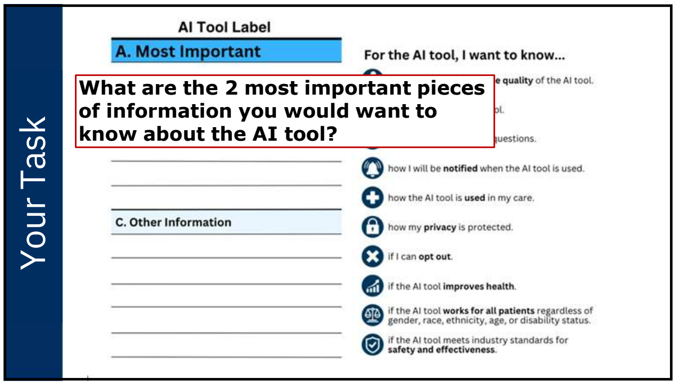

79

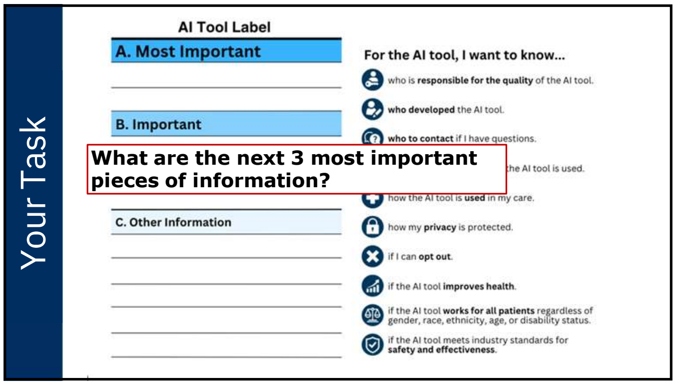

80

Breakout Room Discussion Overview

- Individual Label
  - What are your own priorities? Important? More important? Most important?
- Small Group Label
  - What are the priorities of the small group?
  - How can we make the label better?
  - Will it improve patient understanding?

81

Q&A

Questions for the presenters

UNIVERSITY OF MICHIGAN

82

Today's Agenda

9:00 - 9:30	Welcome and introductions
9:30 - 10:15	Session 1 presentation: AI in Healthcare
10:15 - 10:25	Session 1 Q&A
10:25 - 10:30	5-minute break
10:30 - 11:30	Breakout Room discussion: Icebreaker & Perspectives on AI
11:30 - NOON	Break for Lunch
12:00 - 12:45	Session 2 presentation and Q&A: AI Policy
12:45 - 1:45	Breakout Room discussion: AI Tool Label Activity
1:45 - 1:50	5-minute break
1:50 - 2:30	Large group discussion & review

83

Breakout Room Discussion

AI Tool Labeling Activity

UNIVERSITY OF MICHIGAN

84

Today's Agenda

9:00 - 9:30	Welcome and introductions
9:30 - 10:15	Session 1 presentation: AI in Healthcare
10:15 - 10:25	Session 1 Q&A
10:25 - 10:30	5-minute break
10:30 - 11:30	Breakout Room discussion: Icebreaker & Perspectives on AI
11:30 - NOON	Break for Lunch
12:00 - 12:45	Session 2 presentation and Q&A: AI Policy
12:45 - 1:45	Breakout Room discussion: AI Tool Label Activity
1:45 - 1:50	5-minute break
1:50 - 2:30	Large group discussion & review

85

LARGE GROUP REVIEW

AI Label Report Outs

UNIVERSITY OF MICHIGAN

86

Large Group Overview

- Facilitators share Breakout Room Labels
- Large Group Discussion
  - General thoughts and impressions about labels
  - Questions or concerns?
  - Will it improve patient understanding?
- Wrap/Up and Feedback

87

THANK YOU

Please fill out Survey 2! You will receive the survey link shortly.

UNIVERSITY OF MICHIGAN

88

Appendix Table 1. Supplementary quotes from small group discussions

**Themes**	**Definition**	**Exemplary Quote(s)**
**Hopes for the Future**	Expectations for AI to improve healthcare for current and future generations.	I'm excited about the possibilities of curing, say, diabetes and hypertension. […] Perhaps we can get rid of all of these diseases that seem to elude healthcare right now. (Deliberation 1)<br><br>I'm excited because of the groundwork that's being put in now around AI, and what it could possibly look like for my grandchildren and my great-grandchildren. (Deliberation 1)<br><br>Because it's the future. Our kids are going to grow up and get older and you want them to be able to do things that we couldn't do. (Deliberation 2)<br><br>It would be wonderful if children would not have to die from diseases that they don't have cures for now. Cures that are developed through the use of AI. That would be wonderful. (Deliberation 2)<br><br>I'm just excited about what technology is doing and where it's moving us forward in regards to our health. (Deliberation 2)<br><br>I'm excited about just technology and the quickness of technology and the accuracy. If ChatGPT is an indication of where we're going, it is amazing, where you can take a couple of items, and it'll write a whole research paper. It does all your marketing, and so if we could do that in the marketing area, what our health looks like is going to be amazing. (Deliberation 2)<br><br>In 10 years, it's going to be something like a calculator. I don't think anybody can anymore solve a math equation without using their calculator at this point. I do believe it's going to be one of those situations where it just exists and we accept it. Like the internet, like our cell phones, like anything else. (Deliberation 3)<br><br>I'm excited because I do think that there is the possibility of more breakthroughs in healthcare. I think that they will be able to analyze data a lot better. Once they do collect it to come up with cures for more diseases that haven't been cured. (Deliberation 4)<br><br>I think another benefit for my family, you know, grandchildren and that kind of stuff is what we're doing now is absolutely going to benefit them. Hopefully they won't be the guinea pigs. You know, we're just collecting data. We're starting to get it together. AI is going to be big. And I would assume as my grandchildren come up, they will be benefiting from it greatly. (Deliberation 4)

**Information Access and Efficiency**	Expectations that AI will facilitate more convenient and rapid access to healthcare information for patients and providers	I think that one of the biggest benefits that we have with AI is the amount of information that's accessible to the doctor on a moment's notice [...] That is something that really will be a benefit to the medical community, and myself as a patient. (Deliberation 1)<br><br>For me, the excitement is just the access to the amount of information that potentially could be helpful in comparison, and statistics, potential treatment and things like that. It's just even now when I'm looking for something, I could just go on the web and find tutorials and things like that. I'm not saying that that would be the same in healthcare, but if they had any questions about somebody in my age range with some of the conditions or anything like that, that it's just available so quickly that there is not a lot of time and lapse in information access. (Deliberation 1)<br><br>I do see where having all of that information at your fingertips would better assist the physicians and clinicians to make a good assessment. (Deliberation 1)<br><br>I feel like AI would be a lot faster. It's quicker. The AI gathers the information, and it's quick. (Deliberation 2)<br><br>For me, the benefits are great as far as it came to healthcare. You can get diagnosed faster. You can get help faster. (Deliberation 2)<br><br>I'm excited about just technology and the quickness of technology and the accuracy. (Deliberation 2)<br><br>I like the simple fact that AI is coming in, because computers think more quicker and faster and can retain more information than the human person. (Deliberation 2)<br><br>I just think it's so cool and I think it just saves a lot of time. [...] someone can build something that's not a actual human talking to you that can give you a diagnosis or it can do multiple things, like answer a question so fast, give you a paragraph about something you're asking or a picture for you all within five seconds. I just think it's very interesting and I think it's amazing. (Deliberation 3)<br><br>I think due to time constraints of physicians, doctors, surgeons, and their staff, I think as a patient, you can maybe, dig deeper into information you've been given on your own without having someone stand there and try to tell you or to put it in a language or a set of terms that you can understand. With AI and some of my experiences, I start out by saying, explain this to me like I'm a five year old. What are the benefits of whatever? [...] current schedules don't allow for the human doctor to actually sit there and chat with you over a cup of coffee… (Deliberation 4)

		I found here lately that a lot of doctors carry phones with them during the visits. And I had a lot of questions that he didn't have the answers for at the time, and he was able to go onto his device and give me a lot of answers that, he may not have been able to get back with me on right then and there at the appointment. So I thought that that was a really good tool that he had there. (Deliberation 4)<br><br>It makes for a more efficient visit from the doctor because they get all the information they need up front. […] And then that, in my opinion, has left more time for my patients to then unpack their concerns with the doctor and whether or not the doctor listened to a whole other conversation, but it does create more space for there to be more time for individual concerns. (Deliberation 5)
**Advancing Clinical Care**	Expectations that AI should support more accurate, timely, and equitable diagnosis, monitoring, and clinical decision-making.	I feel pretty excited about how to use the data from the X-ray and the MRI because I believe if they collect enough data, it probably can decrease the human error to diagnosis the disease. (Deliberation 1)<br><br>One of the things that stood out for me is the potential for a better diagnosis. (Deliberation 1)<br><br>I believe there are several diseases that are underdiagnosed by doctors. Something like this might be able to better diagnose based on the statistical information that's available by the application. (Deliberation 1)<br><br>I am pretty excited about [...] glucose monitoring and stuff, which I think could be very helpful. Or substance monitoring, I think, could be very helpful because both those things are things that can be very dangerous if they're out of control and if they're not monitored correctly. (Deliberation 1)<br><br>Artificial intelligence can now with 3D imaging took impressions of my teeth and my mouth and made a crown that is perfect. […] The perfection involved, there is no error at all. [...] Like doing an operation or a procedure, it would be perfection. There would not be any type of hesitation or insecurity for the doctor. He wouldn't be thinking about something that happened the last time he did this procedure. (Deliberation 1)<br><br>I would think the system would be responsive to recognize mistakes and the impact it might have and adjust for that, go back to histories, things like formaldehyde babies. How many babies were born with birth defects after the fact until we recognized that that drug caused those outcomes? I think the response of the system with AI would be much quicker to diagnose the mistake and take corrective action. (Deliberation 1)

		I have had poor healthcare from a physician [...] Things about the healthcare team that are deficient can be overridden by the AI. That's the benefit I would imagine. No matter how tired the doctor is, no matter how old, or a language barrier, other things like that might impact their decision. (Deliberation 2)<br><br>AI can touch different areas. It has a vast knowledge. When you go to the doctor now, they're sending you to a specialist. [...] I would just hope with AI that that will streamline the process. That's my pro of AI... It is a quicker, faster diagnosis. (Deliberation 2)<br><br>I just think the [test] results will be quicker, and then that way they can catch and diagnose situations and problems and illnesses quicker. (Deliberation 2)<br><br>I think you shouldn't depend on AI, but you should definitely utilize it. It can be really useful and really helpful. Not only can it detect diseases perhaps a doctor typically couldn't detect, but could do it much quicker, too. (Deliberation 3)<br><br>I think that the most exciting part about the use of AI in the healthcare system is that if an AI tool is well-trained it can help detect diagnosis that doctors might sometimes miss using traditional analysis methods. (Deliberation 3)<br><br>I think this is part of where AI might be helpful in the sense of flagging [cancer] maybe for a physician so that they can do earlier screenings. (Deliberation 3)<br><br>I know there's already been a bunch of breakthroughs recently. With it is the pattern recognition stuff like cross-referencing different studies, data finding like I don't remember the specifics, but there was one about how they had found some new breakthrough with migraines and how to treat them because an AI system had cross-referenced a bunch of different studies. That stuff, I find, is exciting and not nearly as problematic... (Deliberation 4)<br><br>I would say the information about cancer and having data about a lot of different people. [...] I think that's what I'm most excited about is making cancer less prominent in healthcare.[...] I feel like if we had better health tools to diagnose it, to treat it, to, find it or do testing to prevent before it's too late is what I'm really excited about, finding it before it happens, or finding the percentage of how likely you are to develop something... (Deliberation 4)<br><br>And if AI is maybe monitoring the vital signs, maybe they could pick up on something and alert the nursing desk immediately quicker than, you know, a nurse actually monitoring themselves. Because sometimes a nurse or

		someone is not at the front desk because they're out changing patients, helping patients to the bathroom or anything. So that may be a good time to use AI. (Deliberation 5)<br><br>I think the benefits [of AI] would be more comprehensive diagnosis instead of just one person looking at your situation… (Deliberation 5)
**Streamlining Administrative Processes**	Expectations that AI will reduce burdens related to scheduling, paperwork, and wait times, leading to smoother patient experiences.	I could be excited that rather than make judgments about asking a question of my doctor and going through the portal and seeing the documentation the way it's being billed and paid for, to see a question answered by a bot and go through the system and be half the cost or a tenth of the cost of my talking to the doctor. (Deliberation 1)<br><br>The benefit is the scheduling… (Deliberation 2)<br><br>…when it comes to the front desk, they're using AI to schedule appointments. It's helping them organize things more efficiently and faster than before. […] it's making the office and everything run more smoothly than it did from before. (Deliberation 3)<br><br>Every time I go to the doctor or the urgent care, the waiting time is so long. Especially if you're going to the emergency room. I remember the last time I was in the emergency room, it took me five hours to get in. I'm excited to see how fast or how fast they can get the appointments going or how long the waiting time is going to be with the use of AI. (Deliberation 3)<br><br>It can make certain things people don't want to do or tedious things really quick and efficient. (Deliberation 3)<br><br>I see a benefit to having AI in healthcare I think more from an administrative sense. Maybe a more efficient way to create appointments, a more efficient way to confirm appointments with patients so that you don't have any missed appointments. Things like that. Maybe sending them surveys, using AI to make surveys or that pre-appointment questionnaire more accessible if they have linguistic barriers. Things like that. (Deliberation 3)<br><br>… it makes a lot of jobs easier for people, especially when hospitals or health care, places like that really busy. I think it can help in many ways. (Deliberation 3)<br><br>Probably the time that the doctor is in the room because he'd be able to speak to [the AI tool] like they said in all of his notes and stuff. You won't be in there too long, because sometimes we sit in the waiting room for 2 or 3 hours and he'll be in the room, or she'll be in the room with their patients a long time. So it is shorten the wait time of being in the room. (Deliberation 4)

		It'll definitely help timewise, with me doing charting because it usually takes me, depending on a client, it could take me up to an hour to chart, whereas if I'm using AI, it may take like 15 minutes. (Deliberation 4)<br><br>Some of the tools that I'm excited about, and you've heard me mention are autonomous tools for scheduling or order reminders. [...] Let's say you're a diabetic and you need an eye exam because diabetics can develop retinopathy. And often folks don't remember that they've been asked to go get an eye exam. So things like that help, remind and motivate us to complete what the provider has ordered. (Deliberation 4)<br><br>…generally speaking, healthcare is administratively burdened, and it costs us as consumers and as taxpayers, quite a bit of money to pay for a system that pushes paper and, you know, spends time transcribing when we would rather be paying for that time spent directly with us, the consumer or ask the patient. So some of the benefits, to me the autonomous AI examples best apply in the administrative issues. So claim generation, claim processing claim up from the encounter to the insurance company scheduling reminders. (Deliberation 4)<br><br>…you can use AI to speak many different languages, which is really cool. So it can help with getting marginalized groups care and be able to speak their language as well. I'm a therapist, so I'm more on the personal health care, mental health care stuff. And so we have used AI for writing case notes. It saves so much time. (Deliberation 4)<br><br>I'd be excited if they could figure out a way where I can schedule an appointment without having to call and wait on hold for 20 minutes to talk to somebody. (Deliberation 5)<br><br>I do like AI being used to record meetings and things with the doctors. The doctor doesn't have to sit there and take notes. He can focus on what's going on and then doesn't have to worry about things being forgotten. That's already being taken care of. (Deliberation 5)
**Transparency**	Expectation for clear, accessible information about when, how, and why AI is used in healthcare, including details on algorithms,	… transparency, and no one is being surprised when they find out that it wasn't an actual physician that is making some sort of decision for them. (Deliberation 1)<br><br>I would expect that if I walk into my doctor's office, and they tell me they're using a tool, that they have properly vetted what they're using for my care. (Deliberation 1)

	data, risks, and involved parties.	My concerns could be addressed by full transparency. How is it developed? What is the purpose? What is the goal? Who is regulating? (Deliberation 1)<br><br>I think one of the ways that we can address people's concerns is just by making sure that there is a lot of clear information about how AI is being used and how it's being effective, but then also being transparent about potential risks. (Deliberation 1)<br><br>For me, it would help to have it very clearly listed out who's responsible if something doesn't go how we need it or want it to go and very specifically what the process is for addressing it, correcting it, compensating for it. (Deliberation 1)<br><br>… if they do have a glitch in the system that they're making us aware of the glitch. They're making us aware of that they are using AI. A lot of this sometimes is happening, and we're unaware. I just think that's one of the major things that I would want to know… (Deliberation 2)<br><br>I think that the use of AI in healthcare could be very promising, but I also feel like people fear what they don't know, so I think that to get people behind the use of AI in healthcare people should be well-informed about this entire process and this new concept. (Deliberation 3)<br><br>I share similar concerns to others, the main one being transparency because I think as patients, if an AI tool is being used in our healthcare services, I feel like we should know how that AI tool was developed, by who. Also, when it comes to the data I think that patients have the right to know how that data was collected and who the data represents so that we can have a better informed perspective on how that tool is being used in our healthcare service. (Deliberation 3)<br><br>And my concerns would be transparency of algorithms. [...] The transparency of the development of the software that's being used to query and come up with answers from the data. The transparency about collection of data, how that's done. (Deliberation 4)<br><br>…if clients do decide to use AI, are they going to educate them on what all of this means and how it's going to be a benefit and a risk to them? Are they going to be transparent about AI accessing important medical records...? (Deliberation 4)<br><br>The first thing we would need is transparency. Like some sort of information right up front on what's being used, why it's being used… (Deliberation 4)

		I would like to see more news, information. You know, like a short information about a particular topic like on AI so that the general public can get more educated. (Deliberation 5)<br><br>I would like to see more education on what AI is, because before I started this session, I thought of people putting different faces on robots. That's what I thought about. And it wasn't. I really had no clue what was included in AI. And now I realize what it is, and now I know why I get ads for guitars when my husband's on his phone searching for guitars and, it's I think we need more education of the general population to know what AI is because at first I thought, oh, I don't want to do that. That's scary, you know? And then I realized it's already going on. So, I need to know what is in existence already and how it's used. (Deliberation 5)<br><br>I think there needs to be an educational aspect […] I think it's a matter of making sure that people that have access to their records or whatever on technology in a portal that they are taught how to use it so that they can access the information that's available. (Deliberation 5)<br><br>Also, who are we going to be able to actually ask personally? So when we're at the doctor, like we've already said, you see your doctor for a few minutes, you're normally with a nurse or an assistant. Are these people going to be trained and able to actually answer your questions [about AI]? (Deliberation 5)<br><br>Maybe at your doctor's office or the doctor that you're dealing with, maybe some kind of portal where you can put in some questions or concerns or, I don't know, something where they can be answered or, maybe some kind of biannual annually, review of how things are going as far as the AI in a particular facility, things like that. […] How it's being used, some of the concerns you may have and things like that. So, questions and concerns can get answered. And we're not just left in the dark… (Deliberation 5)
**Oversight and Regulation**	Expectations for governance, testing, and accountability in AI deployment, with calls for human involvement, public input, and clear policies to ensure safety and trust.	I feel like I would like to see some kind of precedent or very explicit law or regulation […] who is responsible and clearly spelling out what kinds of things they can do with it and what rights the people have to opt in or out and if something goes wrong. (Deliberation 1)<br><br>I think one of my concerns can be addressed by knowing what my remedy is, or what is the remedy to me if something goes wrong. […] in law, when there is a harm done to an individual, the law states that there needs to be a remedy to make things right, or as close to right as possible. (Deliberation 1)<br><br>I think I would like to know when AI’s being used in my care. I think that might affect decisions I make relative to planning my care. (Deliberation 1)

		I just think we should be heard and considered… (Deliberation 2)<br><br>Somebody needs to make sure that there's proper oversight in the protection of our information because my information is out there. […] That bothers me, so now I'm looking over my shoulder all the time to make sure that nothing is being used and things like that. Oversight on the privacy issue is one of my main concerns. (Deliberation 2)<br><br>I think that the people who will be developing should be in on this panel listening to our concerns and addressing them, taking them seriously. […] They need to figure out how to make us more comfortable with that and whether or not that we can trust the doctors as opposed to trusting AI. That's what I think. We need to be taken serious and listened to. (Deliberation 2)<br><br>There needs to be accountability. (Deliberation 2)<br><br>Who's controlling the people that's controlling AI? (Deliberation 2)<br><br>They haven't caught up with AI yet. That's alarming that there are no policies or laws restricting or really wrapping its arms around AI yet. Anything goes like it's the wild, wild West. (Deliberation 2)<br><br>I also think that we should be a part of the process of implementing laws and policies concerning AI and our protection… (Deliberation 2)<br><br>… if the human error happens with the developers or the doctor or anything, that they [should] be punished. […] an AI mistake from the developer, misdiagnosis, or sends out information over the Web, or anything like that. I think it should be some type of policy or some type of law. (Deliberation 2)<br><br>… if we're not a part of that process, then I don't think we're going to trust it completely, not knowing exactly who worked on the project and if they were a part of our community. […] I think just knowing that there are people like us on the team and making these strides in working in AI, I think that would—that's really helpful. (Deliberation 3)<br><br>I feel like the development of AI, open AI and all that stuff developed really quick, which I'm a fan of, I guess. It's nice using it. It's nice seeing it develop, but then at the same time in healthcare, healthcare's a big deal. You don't want to make mistakes […] sometimes it's very hard to trust healthcare. Sometimes you fully trust healthcare and if you fully trust healthcare, then you fully want to trust AI they use and therefore make the changes slow. Take

		their time. Make sure all the educations are covered. Really make sure that there is no mistakes before you just straight up integrate it with all the other—like all the other AIs. (Deliberation 3)<br><br>I'm wondering, is there like a board that does healthcare in AI and they have a written agenda of regulation. What it includes like, is everyone going to be able to access this pamphlet that they bring out and who's they? You know, who is the board? Is there a board of AI? Is there an owner of AI? Is it like each individual hospital has software that they use in lieu of having AI? I'm just wondering, like, is it a legislative thing? You know, there's just a lot of things that I think about, when it comes to the structure of it in our health care. (Deliberation 4)<br><br>How should it be addressed? Well, it's multi-layered. It's just like what we do now, right? You can make complaints to the office or the health system if those aren't addressed. You can make complaints to your state licensing body. (Deliberation 4)<br><br>I'd say the biggest risk is people's lives are on the line. You know, if I get lost in the woods [using GPS], I might be okay. But if a health decision is made poorly, or if it's not caught in time, people can get hurt. (Deliberation 4)<br><br>I am interested mostly in responsibility and the chain of development and who checks and double checks, along this chain of information […] from diagnosis through the AI systems and then back to the patient. My concern there, basically is everyone in that chain needs to be responsible for the data that's coming and going and being presented back to the patient. (Deliberation 4)<br><br>I would want is that there's still a human reviewing the information and that people's information is protected since, I get emails from my work security team. So, I hear about basically every data breach that happens and I've had my data compromised by multiple breaches this year. So, like anything we could do to decrease the risk would help with me feel more confident with it. (Deliberation 5)<br><br>So it all comes down to who's creating that algorithm. Like when you do a Google search, they can create an algorithm where you see this post and this post first and it totally washes out other posts. So it's who regulates the person who creates the algorithm is the most important part. (Deliberation 5)<br><br>The stakes are just so much higher. Like you can drive somewhere else if you get bad directions, but if you're misdiagnosed, that could be your life or years off your life. (Deliberation 5)
**Human Role**	Emphasis on maintaining human involvement and control in healthcare decisions, with AI	I'd also like to know whether they was completely going by what the AI said or was there several people that's going to review what the AI said. That's really important because I think, in some cases, they might totally just depend on AI and go with what the AI says. That's frightening to me. (Deliberation 1)

	positioned as a supportive tool rather than a replacement, to preserve human judgment, empathy, and patient-provider connection.	It is nice to have a second perspective or to have AI help support or negate something that a doctor already believes. It can act as a second opinion instead of just being the main opinion. That's something that I could be excited about. (Deliberation 1)<br><br>I think my biggest concern again is the decision-making it has. When the AI model's making these fringe decisions, how it weighs those different factors. Also, essentially, I think the way it should be addressed is that the patient, or whoever is representing them, is always making the final decision… (Deliberation 1)<br><br>AI can benefit as a complementary tool, not a substitution because being human means, at some points in time, some mistakes are inevitable… (Deliberation 1)<br><br>I'm thinking that AI is just a resource in the doctor's arsenal of equipment to administer appropriate and good care to his patients. (Deliberation 2)<br><br>…if you are diagnosed with some type of artificial intelligence device or whatever, that it's followed up with a primary physician to say yes, that's the right. (Deliberation 2)<br><br>AI and the doctors [should be] working together, not just going by the data of AI but also the doctor looking at it and researching it and making the best decision for our health. (Deliberation 2)<br><br>When I go to the doctor, I want to make sure he knows all about me before he makes a decision of how to treat a problem I brought to him or her. It was interesting to find out that AI could possibly aid in this process. I think the human element is always important though, because you just can't go through life looking at facts and coming to a conclusion. I think you have to have some reference to your experience in life yourself. When I had COVID, it helped me to understand what COVID was about. Because my doctor had had it, and a machine's not going to have COVID to know what I'm going to experience when I have COVID. You see what I'm saying? (Deliberation 2)<br><br>Well, the benefits to me are very obvious by the fact that the computers can access and interpret much more information than a human being can. It's just one tool for the doctor to use, one tool. The doctor still has to integrate all the other information he has learned and experienced over the years, benefited from the experience of his peers and coworkers. It's available to him. It offers him something else to come up with the right answer for a problem. (Deliberation 2)<br><br>You shouldn't just develop a great AI model and just completely depend on it because then that lack of human trust, that lack of human relationship, interaction, that disappears and we don't want that. You don't want to

		totally depend on the AI because if anything goes wrong with it or whoever is behind the scenes with AI can just do whatever he or she pleases. (Deliberation 3)<br><br>Because me personally, let's say I am going to a physician who is incorporating AI to help him with diagnosis, I'd want a second and third opinion before taking whatever he says to heart. (Deliberation 3)<br><br>A human being has to be involved in the testing and looking into things because I don't know how can we a hundred percent rely on AI. I feel like it's something good that it's going to benefit a lot of people, especially with diagnosis and imaging testings, but a human has to be there just catching little mistakes, especially something still in the process of developing. It's not hundred percent there yet. (Deliberation 3)<br><br>The human factor should always be there when it comes to the healthcare system. At least when a doctor's delivering bad news to a patient, the human empathy should be there. The human creativity should be there. (Deliberation 3)<br><br>For me, AI should just stay assistive. I'm not against AI at all, but it should stay assistive. If you delete them, the human interaction, you're going to delete the whole humane aspect. Medicine is a practice that has to do with human interaction. Deleting that is going to delete—it's going to be automated so there's no compassion in it. (Deliberation 3)<br><br>I trust the human, for me. For now. I don't know, maybe 10 or 20 years later, I don't know. Maybe we will trust the AI more than the human. (Deliberation 3)<br><br>Some of the autonomous solutions that I hear people fearful of is like when it has decision making authority. […] Well, the question that everyone's having is, should we listen to the AI tool, or should I listen to my training? And if I make that decision and it ends up having an adverse outcome, who's to blame, the AI tool or the provider? And so autonomous use of AI seems more applied in the administrative examples. But the assistive tools are really helpful in decision making. (Deliberation 4)<br><br>…when you're sick and you're calling in, you don't. I'm just using myself as an example. I don't want to talk to a phone tree. I don't want to hit one for this and three for… I need a person. I need to hear somebody on the other end when I'm in distress. (Deliberation 4)<br><br>It's kind of scary to me that you could take the human component out of healthcare. I just feel like everything in the world is turning more technology based and that does take the human component away from it. And I just worry, I guess, about everybody being put in a big pot and, you know, everybody's different and unique. So I don't think one cookie cutter way of doing things is going to work for everybody. I just don't see that, working in the long run for everyone. (Deliberation 4)

		I am not so much concerned about AI in health as long as there is a human interaction, along with the affirmation that the AI is helping to diagnose a problem. I don't think it should be necessarily the first step in diagnosis, but I do think that it's a tool that can be used so that it's, I would feel more comfortable having. It's like having another set of eyes that are taking care of you. (Deliberation 5)<br><br>I just want to make sure it's a physician or a nurse or someone there that's priority. And then maybe AI is used as a second source. And that's it. And I don't want to get to the point where in the hospitals or doctor's office offices, they're using that as the primary source… (Deliberation 5)<br><br>I'm wondering how information… in terms of tone of voice, posture, a number of things would be available to AI? If not available, how that might produce wrong information? So, I think a tendency in us is always to question it, and that's a good thing. (Deliberation 5)<br><br>I already feel like doctors […] they can't even tell me by name, you know what I'm saying? And I feel like introducing AI would create more of an impersonal imbalance between doctor-patient relationship and break that down even more than it already is. (Deliberation 5)<br><br>…one thing I really worry about with it is that doctors are going to depend so much on the AI [that] they don't pay attention to what's actually going on with you personally. (Deliberation 5)
